# Plasma shielding removes prior magnetization record from impacted rocks near Santa Fe, New Mexico

**DOI:** 10.1038/s41598-021-01451-8

**Published:** 2021-11-17

**Authors:** Gunther Kletetschka, Radana Kavkova, Hakan Ucar

**Affiliations:** 1 Geophysical Institute, University of Alaska Fairbanks, 903 N Koyukuk Drive, Fairbanks, AK USA; 2 Faculty of Science, Charles University, Albertov 6, Prague, Czech Republic

**Keywords:** Natural hazards, Planetary science, Solid Earth sciences, Astronomy and planetary science, Materials science, Physics

## Abstract

The shock exposure of the Santa Fe’s impact structure in New Mexico is evidenced by large human-size shatter cones. We discovered a new magnetic mechanism that allows a magnetic detection of plasma’s presence during the impact processes. Rock fragments from the impactites were once magnetized by a geomagnetic field. Our novel approach, based on Neel’s theory, revealed more than an order of magnitude lower magnetizations in the rocks that were exposed to the shockwave. Here we present a support for a newly proposed mechanism where the shock wave appearance can generate magnetic shielding that allow keeping the magnetic grains in a superparamagnetic-like state shortly after the shock’s exposure, and leaves the individual magnetized grains in random orientations, significantly lowering the overall magnetic intensity. Our data not only clarify how an impact process allows for a reduction of magnetic paleointensity but also inspire a new direction of effort to study impact sites, using paleointensity reduction as a new impact proxy.

## Introduction

**Figure 1:**
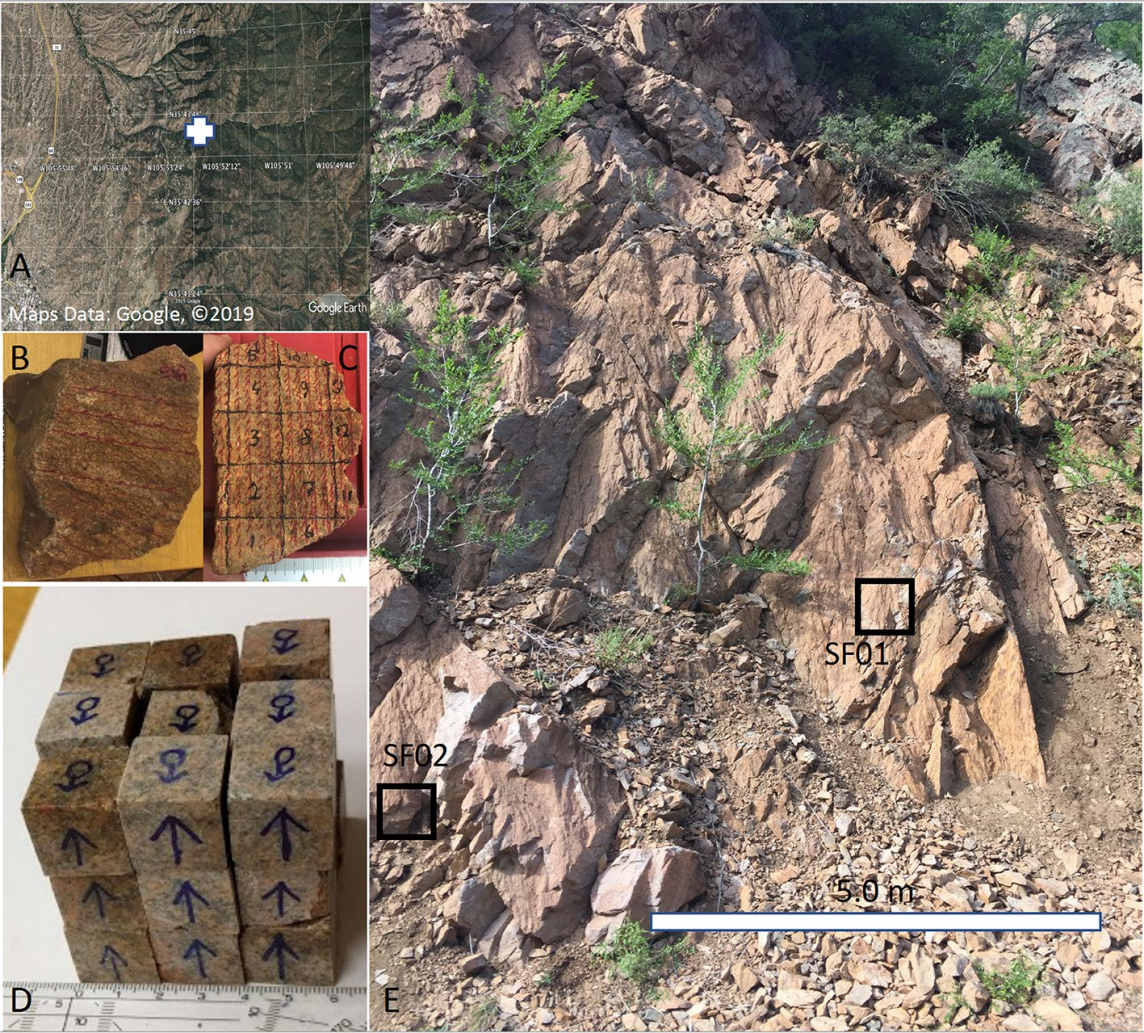
Santa Fe impact structure sample location. (**A**) General location of the Santa Fe structure is just north-east from Santa Fe town, New Mexico, USA. Source: Google earth 7.3.2.5776 (March 5, 2019) Eye alt 20 km. http://www.earth.google.com [March 10, 2019], Map data: Google, DigitalGlobe, Modified in Adobe Photoshop. The white cross is the sample site located at GPS 35°7279 N. Lat., − 105.8613 W. Lon., Santa Fe Country, New Mexico, USA, (**B**, **C**) Rocks SF01 and SF02, respectively, sampled from the metamorphosed granitoid along the road 475 (Latitude: 35.7279, Longitude: − 105.8613) that form part of the Santa Fe impact structure. Rocks SF01 and SF02 are 25 cm and 9 cm across, respectively. Photo by G. Kletetschka. (**D**) Cubes cut from the sample in (**B**). Photo by G. Kletetschka. (**E**) Outcrop containing the metamorphosed granitoid of Santa Fe structure identified by “+” sign in A. Black squares shows the approximate locations of the samples SF01 and SF02. Photo by G. Kletetschka.

The advantage of the Santa Fe structure is that it is a deeply eroded structure that exposes the deep rocks that are not accessible in uneroded impact structures and thus provides a unique evidence of the exposed deep material to the shock pressure wave passing. The eroded deep rock formation (Fig. 1) is metamorphosed Proterozoic granitoid (1.7–1.4 Ga) about 8 km northeast of Santa Fe, New Mexico, USA. Well-developed shatter cones that contain shocked quartz^1^ confirm that this granitoid (Fig. 1B–D) was modified by shock pressure during the impact event^2,3^. While the shatter cones indicate location of a remnant of the central uplift of the impact structure, there is no other supporting morphological evidence for crater features, except for gravity indicators derived from second derivatives of gravity potential^4^, and thus estimates of crater diameter (6–13 km) are poorly constrained^2^ (Fig. 1). Occurrence of zircons in the impacted area place constraints on the age of the impact between 350 and 1472 Ma^1^. Shock planar deformation features were reported from apatite^5^, xenotime^6^ and muscovite crystals^7^.

The motivation of this study was to investigate the effect of impact pressure that produces shatter cones and planar deformation features (5–30 GPa) on Natural Remanent Magnetization (NRM). Near the ground zero, the impact energy pulse would generate high impact pressures and temperatures, where the liquid silica can undergo spike (10^5^–10^6^ S/m) in electrical conductivities^8,9^. At the same time there will be a high temperature plasma, exceeding 10,000 K (depending on the impactor velocity) along the surface^10^. Shock velocities (> 20 km/s) exceed elastic wave velocities of the substrate and result in > 100 GPa overpressure and particle velocities exceeding 12 km/s^9^. However, the pressure dissipates with more distance from the ground zero. The pressure decrease can be modelled by Hugoniot equation^11^, and when applying to large impact structures on Mars, magnetic analysis of rock impacted even with pressure as low as 1 GPa pressure, there is enough energy to demagnetize portion of the original rocks’ magnetic record^12^. Thus, there is an indication that shatter cones and planar deformation features, experiencing 5–30 GPa pressures dissipated from much higher-pressure fields, may be tied with unique magnetic properties. While recent work on impacted material (mostly impact-generated glass) studied primarily induced magnetization^13,14^, the magnetic remanence record may provide more detailed insight into an impact history^15^. Shock effects on the magnetization of rocks and their effects on magnetic anomalies are occasionally discussed^16^ but the rigorous physics explanation behind this phenomena did not go much beyond the classical introductory work on mechanical, short term, effects on rock magnetic properties^17^. Note that the mechanical dynamic shock (e.g. hitting by a hammer), investigated by Nagata, emits seismic waves that generate physically different phenomena than the “real” shock wave that starts near ground zero, exceeding elastic seismic speeds due to speeds of the impactor (tens of km/s). It is the dissipation from pressures > 100 s GPa down to 5–30 GPa that cause the material to uniquely deform, creating features^3^ like shatter cones and planar deformation features (PDFs). We know that rocks were previously magnetized in a geomagnetic field. Information about the paleomagnetic field intensities can be recorded in the samples’ NRM. Rocks from Ward’s collection collected in many geographically contrasting locations showed that, as a general rule, rock are magnetized in geomagnetic field to 1–2% of its saturation level^18^. Elaborated methods were designed to obtain values of rocks’ paleofields by analyzing NRM without heating^19,20^. Rock samples, in general, have their NRMs that depend on magnetic minerals, their grain size, aspect ratio, strain and temperature^20^.

### Non-heating approach justification

The linear relationship in weak fields between the magnetic field and the intensity of thermal remanent magnetization (TRM) suggests that a simple comparison between Natural Remanent Magnetization (NRM), composed of just one component, and a laboratory TRM in a known field can provide, at least within two fold accuracy^20^, an estimate of geomagnetic field intensity in which the rock acquired its magnetization as thermoremanent and/or chemical remanent magnetization^20^. However, there are a couple of catches. First, a single magnetization recording a single event must be isolated. Second, the magnetic carriers of this magnetization must not be irreversibly altered when heated. The classical work by Koenigsberger and the Thelliers soon after WWII laid out the generally accepted solution in terms of progressive double heating methods taking advantage of the law of partial TRM (pTRM)^21,22^, named collectively as KTT). These KTT progressive double heating methods were developed to detect an effect of irreversible changes brought about by heating. However, the problem remained severe and Thellier expressed the opinion that the method would only work with pottery—the ideal material. Since the classic work of Koenigsberger and the Thelliers, there have been numerous attempts to “improve” on their methods, or at least to speed them up. Among those most directly related to the KTT method^23^ is particularly notable. Other important developments utilizing the KTT methodology have been the use of glasses, whose magnetic constituents do not alter on heating^24^ and of single plagioclase crystals with their included fine magnetite^25^, which again does not alter on heating.

Attempts were made to “improve” the KTT method with measurements made at temperature instead of cooling to room temperature to avoid the effect of interactions at lower temperatures, but the technique encountered additional complications because of the variation of saturation magnetization with temperature^26^. Several groups used techniques to check on the nature of the changes taking place during heating^27^. To mitigate the effect of heating Shaw developed his Anhysteretic Remanent Magnetization (ARM) method, which reduces heating times, but still involves a comparison between NRM and TRM^28^. A much more sophisticated approach has been taken to this use of microwaves^29^. However, when these have been compared with standard KTT experiments discrepancies remain^30^.

ARM normalization methods, which involved no heating, were developed^31^. In still another attempt to avoid the effects of heating, it is possible to use the simplest imaginable method of normalization, namely with saturation Isothermal Remanent Magnetization (SIRM)^20^. With the success of this method and its theoretical backing (Supplementary Material) we apply this technique for the Santa Fe rocks.

### Nature of magnetic remanence

There are two processes in crustal rocks that record paleomagnetic information. The first process occurs as the magnetic mineral of constant volume passes the blocking temperature and the fluctuating magnetic moments within the mineral interact with and their alignment is influenced by the external magnetic field (if present)^32^. The second process occurs when the magnetic mineral grows through the blocking volume of homogeneously distributed magnetic dipoles within the mineral, and the mineral begins interacting with the external field (if present) at a fixed temperature^32^. The acquired magnetizations by processes one and two are TRM and chemical remanent magnetization (CRM), respectively^32^. Both of these processes contribute to overall paleofield recording capability with similar efficiency^20^. The methods for paleofield estimates rely on laboratory manipulation of samples by giving them artificial TRM and comparing them with the magnetization originally found. This manipulation, however, may result in irreversible heat-induced alteration^33,34^. Here, we use a normalization approach that uses a two-fold deviation error estimate of geomagnetic paleointensity^20^.

## Material and method

### Material

**Figure 2:**
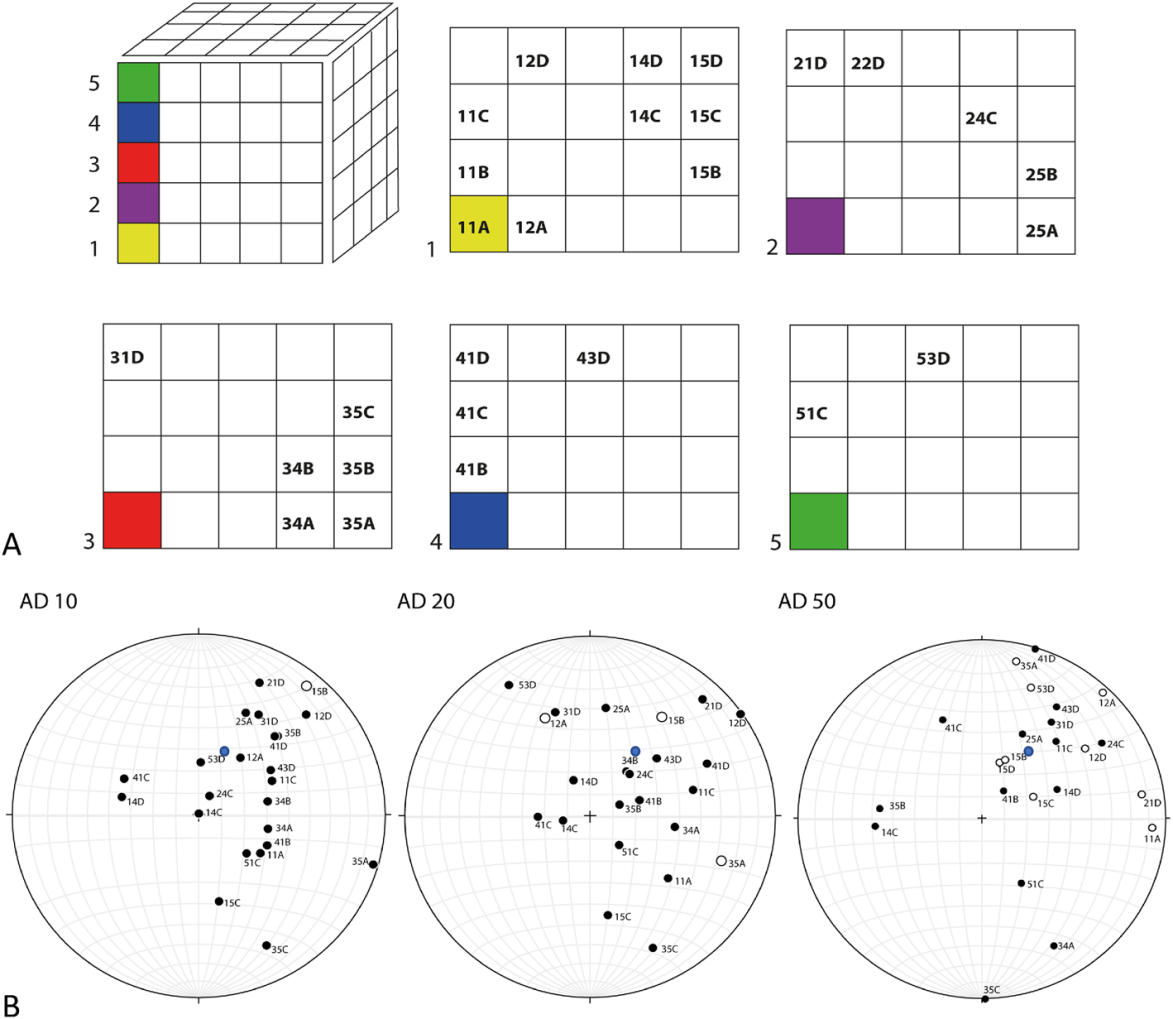
(**A**) Map of the cube labels for individual cubes that survived intact the cutting procedure (many cubes disintegrated during the cutting) of the rock sample in Fig. 1B. (**B**) Stereonet plots are showing orientation of the magnetizations from each of the cube mapped in A. Stereonet plots are labeled by AD 10, AD 20 and AD 50. Plot AD 10 shows each of the cubes’ magnetizations after demagnetization by alternating field of 10 A/m. Plots AD 20 and AD 50 show demagnetizations by 20 A/m and 50 A/m respectively. Blue dots in each stereoplot is direction of today’s field.

We collected several un-oriented rock fragments (SF01–2 kg and SF02–0.5 kg, rock density = 3056 kg/m^3^) from the granitoid that contained shatter cone features (Fig. 1), GPS 35°7279 N. Lat., − 105.8613 W. Lon., Santa Fe Country, New Mexico, USA. Most of the SF01 was used for magnetic remanence measurements while SF02 sub-samples were also used for the curie temperature. The outcrop of shatter cones is part of the roadcut on the local road 475 (Hyde Park Road). Both specimens SF01, and SF02 (Fig. 1B,C) with 100 and 38 subsamples, respectively, were extracted from the location designated by “+” on Fig. 1A. The samples were cut by a non-magnetic, water-cooled saw blade into multiple cubes (8 cm^3^, see Fig. 1). From 100 cubes of SF01 (block of 5 × 5 × 4 cubes, See Fig. 2A), only 27 did not break while cutting due to brittleness of the rock (Fig. 1). Figure 2A shows the map of the individual preserved cubes from the original block SF01 (Fig. 1). Each of the SF01 cubes was marked by two different types of arrows (Fig. 1C), to preserve the respective orientation within the cubes. Each of the cubes weighed about 21 g. Rock SF02 was cut into 5 cubes for Thellier–Thellier heating method (data are not shown due to alteration during heating) and 29 smaller fragments, 4 of which (29A, 29B, 31A, 31B) were used for magnetic remanence measurements (Figs. S28, S29, S30, S31, respectively).

### Methods

Magnetic measuring was performed both in the Geophysical Institute, Reichardt building, in magnetically shielded room and in the Department of Paleomagnetism, Pruhonice Research Centre, Institute of Geology of the Czech Academy of Sciences. We used a non-magnetic plastic holder and measured the Natural Remanent Magnetization (NRM)^32^ using a rotating sample magnetometer JR6 (AGICO Inc.), operating either in the magnetically shielded room (Fairbanks, Alaska) or in the center of a Helmholtz coil system (Pruhonice, Czech Republic), thereby minimizing the ambient field down to 100 s of nT. On several samples we used automatic superconducting rock magnetometer by 2G equipped with demagnetizing coils up to 150 mT alternating field.

Measurements of the effect of change in electric conductivity on geomagnetic field shielding was performed at laboratory of Institute of hydrogeology, geological engineering, and applied geophysics, Charles University. A circular 7 cm in diameter and 1 mm thick plate was made from a high temperature superconductor (HTS) high strength wire with 0.31 mm × 4.2 mm cross section, superconducting at 77 K (American Superconductor). Individual fragments of the HTS wire were cut and made into a 7 cm in diameter circular plate, 0.31 mm thick. Three of such plates were glued together by epoxy forming about 1 mm thick plate, 7 cm in diameter. The plate was inserted into a Styrofoam cup, 7 cm in inner diameter and of 12 cm height with the Styrofoam wall ~ 4 mm thick. This cup was placed on the flux gate magnetometer sensor whose response was monitored while submerging the high temperature plate into the liquid nitrogen (77 K).

#### Magnetic treatment

**Figure 3:**
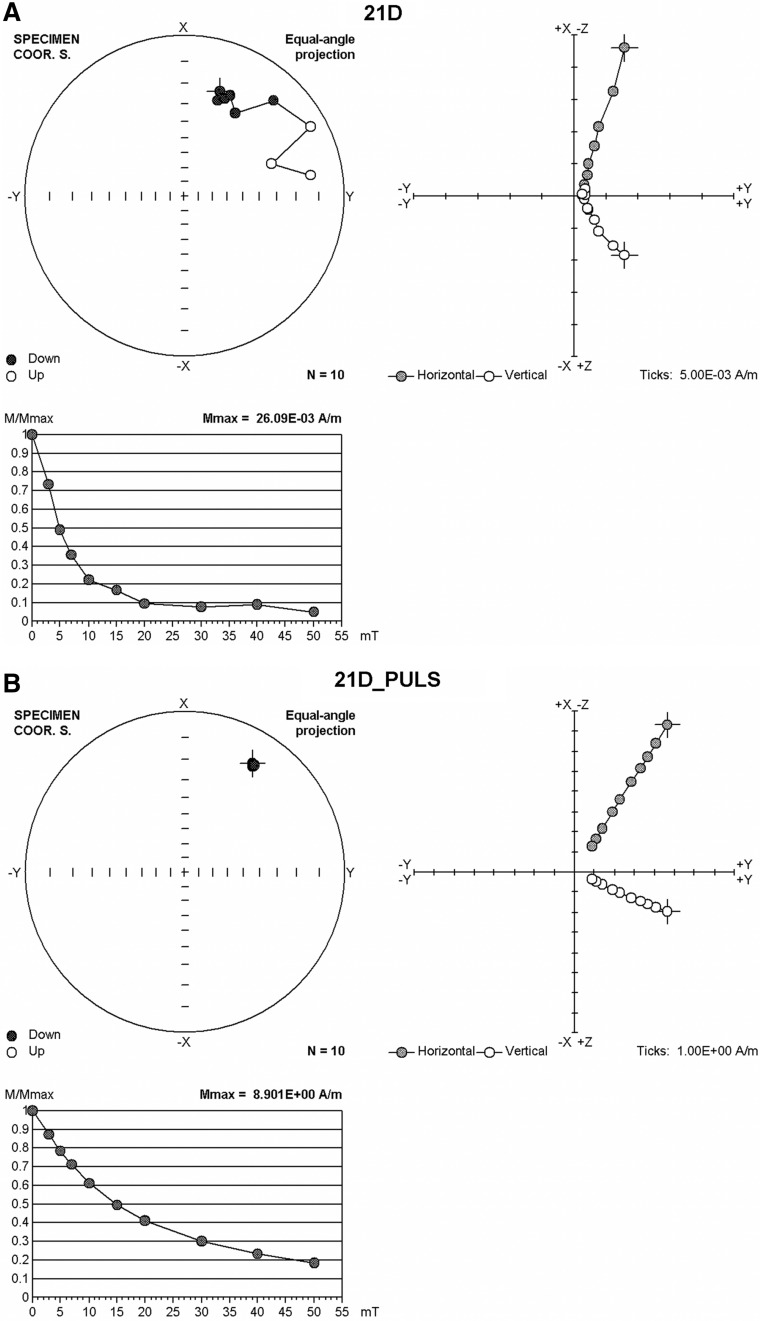
Stepwise demagnetization (3, 5, 7, 10, 15, 20, 25, 30, 40, 50 A/m) of Natural Remanent Magnetization (NRM) of sample 21D is shown in (**A**) in three types of plots. Upper left shows how direction of NRM changed during the demagnetization (NRM + 10 steps, N = 10). Black circle points down, white points up. Cross is the NRM direction prior to demagnetization steps. Upper right is projection of the stepwise demagnetized remanence vector onto horizontal (+ X + Y, gray circle) and vertical (− Z + Y, white circle) plane. Lower left is the stepwise demagnetized magnitude of the remanence vector normalized by stated maximum. Stepwise demagnetization of Saturation Isothermal Remanent Magnetization (SIRM) of sample 21D (labelled 21D_PULS) is shown in (**B**) using the same types of plots as in A.

Each sample was stepwise demagnetized using alternating fields of 3, 5, 7, 10, 15, 20, 25, 30, 40, 50 mT and the remaining remanent magnetization measured after each demagnetizing step. Once the sample was demagnetized by 50 mT and measured, it was exposed to a 1 T pulsed magnetic field at room temperature in the direction of the original NRM (Fig. 3). For magnetic acquisition we used ASC Scientific (Model IM-10–30) with the coil exposing the sample with the magnetic pulse for a few seconds. This process saturated our samples with the maximum remanent magnetization, called Saturation Isothermal Remanent Magnetization (SIRM). Once saturated, samples were stepwise exposed to alternating fields of 3, 5, 7, 10, 15, 20, 25, 30, 40, 50 mT and measured.

### Magnetic mineralogy

Samples from SF01 and SF02 were used for Curie temperature magnetic measurements. We used AGICO KLY4 magnetic susceptibility bridge equipped with the furnace allowing measurement of magnetic susceptibility from room temperature up to 720 °C.

Sub sample SF01, 43D was powdered using agate mortar and Iron Boron Neodymium magnet (60 mm × 40 mm × 2 mm), magnetized in 2 mm direction, covered with polyethylene bag (0.2 mm membrane thickness) was used to extract magnetic carriers from the crushed slurry. Magnetic extract was gravitationally purified to remove larger fragments of mostly lithic minerals that held magnetic oxide grains inside them. The resulting powder was a subject to X-ray diffraction (XRD) analysis using equipment: X’Pert Pro, PANalytical B.V., Almelo, the Netherlands, software: X’Pert HighScore 1.0d, PANalytical B.V., Almelo, the Netherlands, (for details see Supplementary material).

## Results

### Magnetic remanence

**Figure 4:**
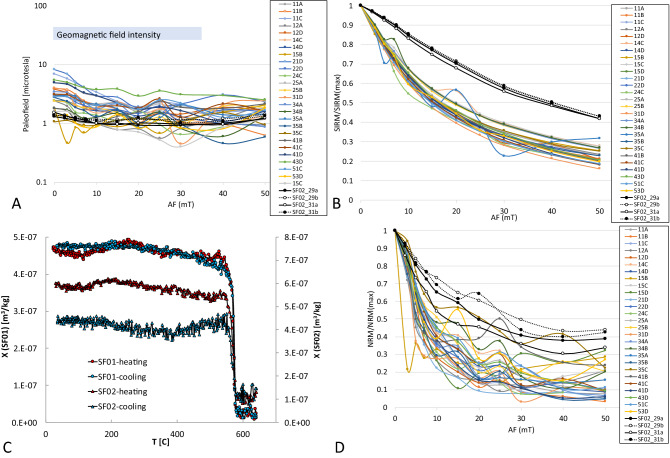
(**A**) Paleofield estimate is shown for 26 samples from SF01 and 4 samples from SF02 that are part of the Santa Fe impact rock containing shatter cone texture. Paleointensity estimates are calculated for each step of demagnetization by alternating field (AF) up to 50 mT. Geomagnetic field intensity range during the last 2 billion of years is labelled and general range indicated by gray panel. (**B**) Demagnetization of SIRM (Saturation isothermal remanent magnetization) is shown for 26 samples from SF01 and 4 samples from SF02 that are part of the Santa Fe impact rock containing shatter cone texture. (**C**) Curie temperature in samples from SF01 and SF02 was estimated by measurement of magnetic susceptibility with increasing temperature. Major drop in susceptibility X near 575 C is consistent with magnetite as the magnetic carrier of these two rocks. (**D**) Demagnetization of NRM (Natural Remanent Magnetization) is shown for 26 samples from SF01 and 4 samples from SF02 that are part of the Santa Fe impact rock containing shatter cone texture.

Measurements of magnetic remanence (Supplementary tables S1–S31) were obtained for both directional stability of magnetic remanence as well as for detection of magnetic paleointensity using Eq. (8). Figure 2 shows a map of the sub-fragment distribution from the original hand specimen SF01 shown in Fig. 1B. These sub fragments were given specific labels that were used in Fig. 2B to show how the direction of the magnetization varies among the sub-fragments using stereonets. We attempted to use double heating method on SF02 samples, but the method was unreliable because the samples altered at 300 ºC. Magnetic remanence at room temperature was examined on four sub-samples from specimen SF02 and summarized in Fig. 4.

The left stereonet (AD 10) shows the direction of magnetic remanence after samples from SF01 were exposed to a 10 mT alternating field to clean them of any possible soft overprints due to handing of the samples. While the relative orientations stay constant within a cone of 5 degrees, AD 10 revealed that neighboring samples have contrasting magnetic directions distributed in a cone with an angle of more than 120 degrees. When the demagnetization of samples was increased using an alternating field of 20 mT (Fig. 2B, AD 20), the distribution increased to cover the opposing hemisphere with a greater than 180-degree cone. Then we demagnetized all samples using an alternating field of 50 mT, and the resulting distribution covered more or less the full 360 degrees, indicating completely random magnetic directions (Fig. 2B, AD 50).

The mechanism of demagnetization of the NRM is illustrated in Fig. 3A for sample 21D. The behavior of all other samples from both SF01 and SF02 is shown in Supplementary Information (Figs. S1–S31). Magnetic behavior during stepwise demagnetization is illustrated in three different plots. The upper left plots in Fig. 3A and B depict an equal angle stereonet projection like the equal area projection in Fig. 2. The original natural remanent magnetization direction is indicated by the cross. Demagnetization by 3, 5, 7, 10, 15, 20, 30, 40, and 50 mT is illustrated by points connected with a straight line. Black points indicate the directions of magnetization point down-ward, and white points indicate an upward direction of magnetization. Note that the first two points (representing demagnetization by 3, and 5 mT) cluster near each other and represent demagnetization by 3, 5, and 10 mT alternating fields. After 10 mT demagnetization the direction is the same, as is shown in Fig. 2B-AD 10 where the data point is labeled 21D. When demagnetization increased to 15 and 20 mT the magnetic direction started drifting towards the horizontal plane in the stereonet. The direction after 20 mT demagnetization is shown in Fig. 2B-AD 20, labeled 21D. The last three exposures of the sample to demagnetizing fields of 30, 40, and 50 mT resulted in magnetization drift towards the reversed hemisphere, indicated by empty circles. The direction after the 50 mT demagnetization is shown in Fig. 2B-AD 50.

Tracing the direction on the stereonet gives an indication about the change in magnetic direction, while the change in the amplitude of the magnetic remanence is shown in the lower panel of Fig. 3A. Note that magnetization quickly decreased during the first three demagnetization levels (3 mT, 5 mT, 10 mT). While these levels preserve one magnetic direction (upper stereonet panel) magnetization is reduced by more than 50%. While further demagnetization of sample 21D by 15 mT and 20 mT reduced magnetization further (but not as sharply as in previous demagnetization steps), the magnetic direction started drifting from the stable direction. With further demagnetizing by 30 mT, 40 mT, and 50 mT the remaining magnetic intensity of the sample did not significantly reduce. However, the magnetic direction did continue to significantly drift (Fig. 3A).

The information about the direction of magnetization and the remanence intensity can be combined in one diagram shown in Fig. 3A, right panel. Here the demagnetization of the magnetic remanence vector is shown in two overlapping projections, where the upper quadrangle shows + X, + Y, for the horizontal plane, and − Z, + Y for the vertical plane. When these data show straight lines the rock contains one component of magnetization.

After the samples were demagnetized, they were all saturated by a pulse magnetic field of 1 T. and the same demagnetization process was repeated. The magnetic behavior of this induced moment is shown in Fig. 3B, where the upper panel shows the stereonet with the magnetic directions, the lower panel shows the decreasing magnetic intensity, and the upper right panel shows the projection of magnetic vector change in horizontal and vertical planes.

### Paleointensity from magnetic measurements

The two data sets, NRM and SIRM demagnetizations, obtained from the collected sub-samples allow us to apply the Eq. (8) for estimating the paleofield that was recorded inside these samples. Figure 4 illustrates the range of geomagnetic ambient field compared with paleofield estimates from all of the sub fragments. Note that the paleointensity values for SF01 are spread between 1 and 8 µT for low levels of demagnetization (0–3 mT). When samples are cleaned by alternating field of 5–20 mT the paleofield estimates quickly drop down to 0.8–2.0 µT. Further demagnetization does not influence the paleofield estimates; they stay between 0.8 and 2 µT. Subsamples from SF02 do not show these low paleointensity enhancements for low demagnetization field and stay around 1 µT over the entire range of paleomagnetic fields. Figure 4B shows that while samples from SF01 require less than 20 A/m to demagnetize 50% of its saturation magnetization samples from SF02 need more than 30 A/m demagnetizing field to reach 50% saturation level. A similar relation holds for NRM demagnetization (Fig. 4D, Tables S1–S31).

### Magnetic mineralogy

Figure 4C shows an example of detection of Curie temperature in the samples from both SF01 and SF02. Both samples, SF01 and SF02 showed more or less constant magnetic susceptibility that suddenly dropped at 575 C, indicating that the Santa Fe rock contains magnetite. During the cooling, the sample SF01 showed an increase to the same level of susceptibility as it was during the warming. However, sample SF02 showed significantly smaller level of magnetic susceptibility after cooling through the magnetite Curie temperature due to alterations taking place during the heating (Fig. 4C). We performed magnetic extraction of the powdered sample (sample SF01, 43D). X-ray diffraction analysis (XRD) indicated that not only magnetite, but also hematite in approximate ratio 3/1 (23% of hematite to 8% of magnetite from the XRD magnetic extract, see methods and supplementary material for more details) is present in Santa Fe material.

## Discussion

### Remanence destruction

Preserving the magnetic orientation during sub fragmentation of the hand specimen collected from the same shatter cone-bearing rocks at Santa Fe allowed us to analyze the directions of magnetic remanence. While there is experimental and observational evidence that an impact can in some cases generate magnetic remanence acquisition^35^, our directional analyses, which show ~ 120° cone cluster in the direction of today’s magnetic field, indicate that these rocks contain only soft magnetization induced by prolonged residence in a geomagnetic field. When the soft component was removed by demagnetization, the spread of magnetic directions kept widening until reaching a full 360° (Fig. 2B, AD 50). Although this randomization can be achieved by pulverization and subsequent conglomeration this is not the case here as the rock is an equigranular granitoid of Paleoproterozoic age^36^.

Shock by impact processes is discussed in the literature as generating a Shock Remanent Magnetization (SRM)^35,37–39^. It is important to investigate the detail when considering literature on experimental SRM. This is because the term SRM mostly refers to the acquisition of remanence by dynamic mechanical conditions (e.g. hitting rock with a hammer), when rock is modified by experiencing elastic waves with speeds not exceeding the speeds of seismic waves^12,17,40^. In this work we refer to the SRM when rock is experiencing deformations that started from near ground zero with speeds exceeding the seismic wave velocities of the magnetite (7.74 km/s), giving combined rock velocities allowed to be transmitted due to granite properties, the longitudinal and shear velocity are 5.33 km/s and 3.28 km/s, respectively^40,41^. This definition of SRM questions many of the rock magnetic experimental work because spectrum of speeds, that is generated from speeds greater than 7.74 km/s for magnetite^40^, is difficult to achieve.

It has been observed that rocks can be both demagnetized (this work) and magnetized^35,38,39^ by shock. These two cases relate to specific conditions of the impact. The presence of the geomagnetic field conditions during the impact contrasts sharply to magnetic field absence during the impact due to ~ 4 orders of magnitude magnetic field intensity difference (50,000 nT on Earth vs 5 nT in space). It this paper we discuss impact when the geomagnetic field was present but shielded due to conducting layer appearance. For this we have a support from the magnetic remanence absence in the Santa Fe rocks containing shatter cones. It appears from modelling that plasma created by an impact may generates organized magnetic fields of sufficient intensity to magnetize the rocks exposed^39^. However, the absence of magnetization over most of the impact basins on the Moon and Mars does not support this modelling. There was an experimental observation that rock may get magnetized during the impact^17^. However, this magnetization is acquired mostly by low coercivity grains and may relate to isothermal remanent magnetization^42^ or magnetization by localized electric discharges like lightning discharges that are known to magnetize low coercivity component of exposed rocks^43^. Such magnetization would be due to electric discharges between the dust accumulating electric charge due to friction^44^ and would not significantly change the magnetization of the substrate rocks.

In addition to the shatter cone and PDF formation during the overpressure wave (5–30 GPa) exposure, any prior porosity present in the granitic substrate would result in pores’ closures, densification often visible near the impact structures in gravity aspects^45,46^, and energy transfer from the elastic wave propagation into the heat^47^. The process of annealing due to pore space closure is apparent even during the porosity closures when small meteorites are decelerating into the earth’s atmosphere^48^ and may have contributed to the temperature increase during the shatter cone formation. Such temperature increase, however, would not be over the Curie temperature of magnetite or hematite due to absence of significant magnetic remanence in these rocks.

Figure 4A shows that low coercivity grains in the rock have slightly enhanced paleointensity estimates over the higher coercivity grains for SF01 samples and does not show in SF02 samples. Assuming that the SRM in experimental work was never acquired for speeds exceeding the seismic velocities we can’t use any data demonstrating SRM acquisition. In the Santa Fe samples, we clearly see enhancement for low alternating field (AF) for specimen SF01. We don’t see the same enhancement for SF02, and more importantly, the overall paleointensity estimates are more than order of magnitude lower than geomagnetic field. For this reason, we consider the magnetic enhancement in low AF fields due to effects of viscous magnetic remanence acquisition for the low AF levels.

Regarding the magnetic carrier of Santa Fe rocks, we show in Fig. 4C clear identification of magnetite based on the Curie temperature of 575 C. Note that while SF01 was reversible during the temperature cycling, sample SF02 was not. This justifies our non-heating approach for paleointensity estimation.

### Shock and viscous magnetic remanence

We see in Fig. 4 detection of slightly larger paleofield, for sample S01, when magnetically cleaning with alternating magnetic fields smaller than 10 mT (Fig. 4A). We don’t see this enhancement for sample SF02. While such enhancement in SF01 indicates that viscous magnetization in SF01 samples, both samples show very low level of paleointensity. The viscous component affects the soft magnetic component^32^, and would be parallel to the present ambient field. The SRM, in low shock velocities, is directed parallel to the field present at the time of impact^37,49^. While both viscous and shock components may be present in combination, as shown in Fig. 4A, they would affect only the low coercivity grains, which show vague clustering (Fig. 3B). The high coercivity magnetization would remain intact, as indicators of shock demagnetization, potentially by impact. This coercivity magnetization feature is apparent in the rock fragment S02, where the low coercivity grains do not have similar enhancement as for S01. This is because magnetic carriers in S02 have, in general, larger magnetic coercivity and therefore, the SIRM demagnetization plot in Fig. 4B significantly differs from the coercivity of the S01 magnetic carriers. Despite the difference in magnetic carriers in S01 and S02, the paleointensity estimates for both rocks show more than order of magnitude lower record of paleointensity (Fig. 4A).

### Comparison with Vredefort impact structure

Vredefort impact structure is one of the largest craters on Earth and contains granitoid material with reported random magnetization characteristics^15,50^. However, more detailed analyses on Vredefort’s shocked material suggested that what was originally thought to be the SRM characteristics, was Lightning Remanent Magnetization (LRM) generated by lighting discharges^50^. This idea was later confirmed by new additional measurements that showed not only that LRM is present in the Vredefort granitoid, but also that the LRM was acquired after the Vredefort rocks were thermally magnetically reset^51^. The Vredefort report^50^ shows randomization of magnetic moments on cm-scale that, in principle, has similar characteristics to what we observed in the Santa Fe rocks (Fig. 2). However, there is one important difference between the Vredefort magnetizations and the magnetizations of the Santa Fe material. In principle, the lightning remagnetization is a unique feature that is known to magnetize rock with its own strong magnetic field, which can come close to magnetic saturation, and thus by orders of magnitude more than magnetizations acquired by exposure to a geomagnetic field^43^. Our data show that magnetic remanence saturation (M_rs_) exceeds the original magnetizations of the Santa Fe material by more than 2 orders of magnitude (SI), and therefore LRM cannot be present. While other types of remagnetization (e.g. chemical, thermal) can add new components of magnetization that could generally lower the original magnetization, such mechanisms would reduce the magnetization by a few percents and have consistent magnetic direction change in neighboring samples^32^. The Santa Fe material, however, contains random directions (Fig. 2), and extremely low magnetization level, which indicates that chemical re-magnetization is unlikely.

Additionally, at Vredefort, the impact was at 2.0 Ga, and the region since experienced thermal metamorphism at ~ 1.0 Ga (Kibaran orogeny). Many mineral chronometers (e.g., zircon, others) in Vredefort rocks show Pb-loss during Kibaran orogeny^52^. In terms of the Santa Fe structure, its age is largely unconstrained^1^, bracketed it from 1475 to 300 Ma), and it occurs in a complex geological setting. The rocks could easily have experienced both pre- and post-impact alteration.

### Superparamagnetism frozen in time interpretation

The directional randomness observed in our samples and substantial decrease in magnetic intensity is consistent with superparamagnetism frozen in time^53,54^. The shock wave provides energy that exceeds the energy (> 1 GPa for magnetite^12^, > 50 GPa for hematite^55^) required to block the magnetic remanence within individual magnetic grains^32^. After the shock wave leaves the material, it blocks the magnetization in randomized directions.

Magnetic directions that were acquired by processes other than SRM or LRM must have been uniform due to the presence of a uniform geomagnetic field for more than two billions of years in the range of tens of µT^56^. Here we propose that the observation of the significant randomness of magnetic directions can only be achieved by the passing of a shock wave from an impact process, which facilitated destabilization of magnetic remanence blocking, and freezing the remanence of individual grains in random directions. This effect differs from the rocks that experience dynamic elastic wave (e.g. from hammer). Exposure to such elastic waves may add a magnetic component that is proportional to piezo-remanent magnetization in the direction of the geomagnetic field and the rocks acquires magnetization^17^. We hypothesize that when the speeds of the shock waves dissipate from speeds exceeding elastic (seismic) wave limits down to seismic speeds, in addition to the conditions met for formation of shatter cones, the magnetic grains would experience a short time lasting compression of the lattice, lowering their magnetic microcoercivities and turn the magnetic states of the compressed magnetic grains into superparamagnetic states with a short-term lasting increased magnetic susceptibility. This magnetic state resembles the state when the magnetic grain is above its blocking temperature. The magnetic grains are susceptible to align their magnetic moments according to the magnetic ambient field present at this very special moment in time and space, during which the rock erases its preexisting magnetic remanence record. Here are the supporting data for this new hypothesis.

### New hypothesis for acquisition of reduced magnetization during the rock exposure to the shock wave

The incoming shock wave compresses the rock forming minerals into their limits of elastic properties^40^ and this means that the velocity dissipation into the minerals elastic speeds generates an extra energy that would need to convert into the work done on the crystal lattice of these minerals. Work done includes: (1) Brittle deformation, and (2) Temporal magnetic isotropy of the magnetic minerals.

### Brittle deformation

The typical range of conductivity of earth impact substrate materials 10^–5^–10^2^ S/m may significantly go up over this range because of plasma generation by shock within the rocks^8,9^. Shock wave from the impact is an inflating hemisphere that both spread along the surface from the ground zero, and penetrates the rock under the impact, initially with substrate particles’ speeds > 12 km/s. Because of the speed through the material is larger than an elastic deformation, the excess energy acts on the rocks via both fracturing, melting, and heating. In this region the substrate contains quartz and silicates for which there is an experimental and modelling evidence of sharp increase in electric conductivity of quartz up to 10^5^ S/m^8^. Additionally, it appears that a rock, undergoing stress induced friction even in much lower pressures, generates plasma forming amorphization changes in both quartz^57^ and feldspars^58^. Plasma generation within the minerals is also dramatically changing their electrical conductivity even in the rock that experience lower pressures (2–30 GPa^3^) causing fracturing that result in shatter cone formation. Thus, we have two mechanisms of electrical conductivity increase. The first mechanism is an increase of electrical conductivity due to rocks’ conversion into the, melts and vapor^8,9^ on the leading front of the shock wave in contact with the solid medium with the substrate particle velocities > 12 km/s (there are no rock forming common minerals with an elastic velocity exceeding 10 km/s^40^). This shock penetrates and slows down eventually into the elastic regime but still causing significant rock fracturing that is recorded as shatter cone morphology and that is associated with the second mechanism of electric conductivity increase due to friction generated during the fracturing^57,58^. Similar effect has been suggested to originate during earthquakes and were discussed in terms of the seismo-electromagnetic changes^59^. Based on these data we infer the presence of the significant plasma presence along the propagating shock hemisphere that form an expanding shock generated plasma surface (ESGPS). The ESGPS is being exposed to the ambient geomagnetic field and its presence induces sets of electric currents along ESGPS in such a way that it minimizes the ambient magnetic field. This is because free moving ions interact with ambient magnetic field by Lorenz force. Appearance of high conducting plasma in the rock was modelled by substituting the plasma by high temperature superconductor (HTS). When HTS material was lowered to 77 K, the free moving electrons started to obey the Lorenz force and lowered the ambient magnetic field near the HTS in a similar way as impact generated change of the impacted substrate’s electric conductivity would decrease the ambient magnetic field during its existence (Fig. S32).

An electric conductivity change of expanding ESGPS essentially reduces the geomagnetic field in its vicinity, temporarily, leaving lower magnetic intensity in its enclosed volume. The effect of shielding of the geomagnetic field is due to the high conductivity nature of ESGPS expanding both above the surface and penetrating into the impacted substrate. This magnetic shield from plasma above the surface also lowers the magnetic intensity underneath the impact site. Observation of the reduced magnetic field underneath the impact site has been observed during the Tunguska Airburst in 1908 and underneath the nuclear explosion test sites where there is an evidence of lowering of the geomagnetic intensity from a highly conducting surfaces that expand and lower the magnetic field measured on the surface for several hours^60–62^.

### Temporal magnetic isotropy of the magnetic minerals

Energy of the shock wave, would, in addition to brittle deformation energy generating ESGPS deposits energy portion in a form of sudden lattice deformation that would make changes in the easy and hard directions of the magnetic oxide lattice magnetization. Recall that, for example, in magnetite, oxygen anions form a face-centered cubic lattice, where in interstitial sites are Fe 2+ and Fe3+ anions form two sublattices of an inverse spinel ferrimagnetic system^63^. While shock generated pressure can reorient crystallites at ~ 60 GPa pressures^64^, much lower overpressure values (~ 1 GPa) are capable of resetting the rocks’ remanent magnetizations^12^. Pressure wave exceeding 1 GPa thus temporarily modifies the two sublattices dictating the magnetic properties and allowing to temporally establish magnetic isotropy. This is valid for magnetite at ~ 1GPa. However hematite at 1 GPa still preserves significant portion of its remanence^12^. Hematite may still continue to hold remanent magnetism up to 50 GPa where there is experimental evidence for its magnetism to collapse^55,65^. Note that measured samples contain shatter cones that form at pressures 5–30 GPa, thus in theory, some of the remanent magnetism of hematite may be preserved at pressures below 50 GPa. While both hematite and magnetite are present in Santa Fe rocks based on Curie temperature and XRD data, their efficiency for remanent magnetization and its stability would be grains size dependent^66–69^.

In transition metals like iron, orbital angular momentum is quenched because the 3d electrons, which occupy the outermost orbitals, experience an electrostatic 'crystal field' due to neighboring ions in the crystal. These 3D electrons outweigh the electrostatic coupling within the atom^70^. The crystal field establishes preferred directions in the crystal lattice and gives the origin of magnetocrystalline anisotropy, easy and hard magnetization direction, the main driver of the ability to generate magnetization, including magnetic remanence. We argue that our observation of demagnetization of Santa Fe rocks by impact is the outcome of homogenizing the crystalline anisotropy, by modifying the outermost orbitals of the 3D electrons, allowing temporal crystalline isotropy. Such temporal isotropy would allow magnetic grains to behave like superparamagnetic grains. Once the shock wave passes away, rock acquires the magnetization of the ambient field. However, because the ambient field is temporarily reduced by ESGPS, the aftershock magnetization should be significantly reduced (Fig. 4A). There is no other process in nature known to us that would allow such reduced magnetization while being exposed to the geomagnetic field intensity. If this magnetic feature is shown to be present in rocks affected by other known impact events, this feature could become a new impact characteristic and for the purpose of this work we call it “Demagnetization by Shock (DS)”.

## Conclusions

A new method^20^ was used to estimate paleofields from the rocks containing large shatter cones and shock minerals due to a significant meteorite impact^3^. In addition to the observation of random magnetization directions within the oriented samples, we show that each of the individual subsamples underwent DS, and thus contains a paleofield that was reduced by more than an order of magnitude from the intensity that would be expected if acquired by a geomagnetic field^20^. The possible inherited existence of DS within the shocked material has implications for studying magnetism of rocks not only on Earth, but also on Mars and Moon, where many impact craters have magnetic evidence of demagnetization by their impact processes^71^. We show that the magnetic characteristics could be produced due to newly proposed mechanism. We claim that the lower magnetic intensity measured in our rock samples is due to combination of two effects. Effect 1. A transient compression of the magnetic lattice allows to lower magnetic microcoercivities and turn their superparamagnetic states. Magnetic grains temporarily lower their blocking temperatures. This cause magnetic grains to align their magnetic moments according to the magnetic ambient field at this transient time. Effect 2. The effect of shielding of the geomagnetic field due to the high conductivity nature of the expanding plasma sphere expanding both above the surface and penetrating the impacted substrate. This magnetic shield lowers the magnetic intensity underneath the impact site. Observation of the reduced magnetic field underneath the impact site has been observed during the Tunguska Airburst in 1908 and underneath the nuclear explosion test sites. We support these observations by our experimental evidence shown in this manuscript. We use an onset of high temperature superconductivity as an analog of the impact plasma formation during the impact and demonstrate a reduction of the ambient magnetic field. Such ambient magnetic field reduction allows magnetic grains in the substrate, underneath the impact, at the region containing shatter cones, to record the level of partially shielded ambient magnetic field. After the pressure wave leaves the magnetic mineral, it securely records the level of the ambient magnetic field present at the time of plasma magnetic shielding. We propose that we have detected this special moment of magnetic shielding in our rock samples. Rock that experienced the shock wave to be shielded from the geomagnetic field during the time when the plasma is generated due to shock penetrating the rock. This represents a new mechanism and new indicator that can be used for identifying the substrate rock affected by an impact in the absence of diagnostic indicators, such as crater morphology or shatter cones. The degree of DS we found cannot be achieved by regular igneous/metamorphic rock terrestrial processes within the geomagnetic field.

## Supplementary Information

Supplementary Information 1.

Supplementary Legends.

Supplementary Figures.

Supplementary Tables.

## Data Availability

All materials, data and associated protocols can become available upon request.
