# Supplementary material for: Plasma shielding removes prior magnetization record from impacted rocks near Santa Fe, New Mexico
Source: Sci Rep. 2021 Nov 17;11:22466. doi: 10.1038/s41598-021-01451-8 (PMC8599688; doi:10.1038/s41598-021-01451-8)
Supplement: Supplementary file 1 — Supplementary Information 1. [file 41598_2021_1451_MOESM1_ESM.docx]

Plasma Shielding Removes Prior Magnetization Record from Impacted rocks near Santa Fe, New Mexico

Gunther Kletetschka ^1,2*^

Radana Kavkova^2^

Hakan Ucar^2^

^1^ Geophysical Institute, University of Alaska Fairbanks, 903 N Koyukuk Drive, Fairbanks, AK, USA

^2^ Faculty of Science, Charles Univ, Albertov 6, Prague, Czech Republic

*Gunther Kletetschka, kletetschka@gmail.com, corresponding author

*ORCID*:

Gunther Kletetschka: https: //orcid.org/0000-0002-0645-9037

Radana Kavkova: https: //orcid.org/ 0000-0001-5251-0406

Hakan Ucar: https: //orcid.org/ 0000-0002-2844-0118

Theoretical basis for paleointensity estimates

Here we provide a short summary of the approach described in detail in our previous work ^1^. The TRM of the Santa Fe granitoid is recorded at the blocking temperature T_B,_ when a magnetic mineral cool below the Curie temperature T_C_ in an applied field H_0_. The critical aspect of magnetic remanence in this method is the ratio of thermoremanent magnetization M_tr_ to saturation remanent magnetization M_rs_ of a single domain (SD) grain of volume *V*. Such a grain has its own unique mineral-dependent saturation magnetization M_s_ and variable coercive force H_C_ ^2^ that is largely a function of mineral composition, shape and stress distribution and is given by

$\frac{M_{tr}}{M_{rs}}=tanh\frac{\mu_{0}VM_{s}\left( T_{B} \right)H_{0}}{kT_{B}}$, (1)

where $M_{rs}$ is the saturation remanent magnetization, $\mu_{0}$ is the magnetic vacuum permeability, and *k* is Boltzmann’s constant. Our prior work ^1^ revealed that the ratio of thermoremanent magnetization to saturation remanent magnetization is independent of magnetic domain state and referred to as the magnetic efficiency

$\epsilon=\frac{M_{tr}}{M_{rs}}$, (2)

which is always less than unity. Note that based on Néel’s theory ^2^, we have a timescale τ during which the magnetic remanence is acquired at the blocking temperature

$\frac{\mu_{0}VM_{s}\left( T_{B} \right)H_{c}(T_{B})}{2kT_{B}}=ln \left( \tau/\tau_{0} \right)$, (3)

where τ_0_ is approximately 10^-9^ s. For τ = 100 s we have ln(τ/τ_0_) ≃ 25. The logarithmic function ln(τ/τ_0_) is insensitive to the time scale during which the magnetization is acquired. For Santa Fe rocks that may have taken a million years to cool through the blocking temperature, this factor would increase by a factor of two compared to laboratory timescales. Combining equations (1) and (3) gives:

$\frac{M_{tr}}{M_{rs}}=tanh\frac{2ln \left( \tau/\tau_{0} \right) H_{0}}{H_{c}(T_{B})}$. (4)

Thermal remanent magnetization M_tr_ is a fraction of M_rs_ (<0.1), (maximum uncertainty of ~25%), and we simplify:

$\frac{M_{tr}}{M_{rs}}\approx H_{0}\frac{2ln \left( \tau/\tau_{0} \right)}{H_{c}(T_{B})}$. (5)

Then equidimensional magnetic minerals with variable M_s_, have field strengths proportional to the product of the magnetic efficiency ε and the saturation magnetization M_s_ ^11^

$H_{0}=\psi M_{s}\left( T_{r} \right)\frac{M_{tr}\left( T_{r} \right)}{M_{rs}\left( T_{r} \right)}$, (6)

ψ is a dimensionless empirical constant, and *T_r_* is room temperature. Combining equations (5) and (6) we obtain:

$\frac{H_{c}(T_{B})}{2ln \left( \tau/\tau_{0} \right)}=\psi M_{s}\left( T_{r} \right)$ (7)

where the constant ψ is related to the magnetic coercivity at the blocking temperature, the saturation magnetization of the mineral, and the timescale that the magnetization is acquired at the blocking temperature. Note that this relation displays insensitivity of the efficiency to domain state and grain size ^1^ and allows evaluation of the constant ψ for the common magnetic minerals. When writing in terms of the magnetic induction B, as opposed to the magnetic field H, we have:

$B_{0}=A \frac{M_{tr}}{M_{rs}}$, (8)

Here the constant A depends on the type of magnetic mineral and corresponds to the minimum magnetizing field by which the magnetic mineral archives saturation remanence.

If the magnetic remanence in the Santa Fe rocks is carried primarily by magnetite, this would allow us to use A=2800 for paleointensity estimates of Santa Fe impact rocks ^1^.

Effect of electric conductivity change on ambient magnetic field

We created a laboratory experiment to see if a change in electric conductivity (production of plasma by shock wave) would have any effect on ambient geomagnetic field. This feature was modelled by placing a high temperature superconductor (HTS) material (1 mm thick plate of 7 cm in diameter, see methods) perpendicular to the geomagnetic field (Figure SM1). When HTS was submerged into liquid nitrogen at time 0 s (Figure SM1), in a few seconds the HTS lowered its temperature to 77K, and HTS generated free moving electrons within the superconducting plate. The free moving electrons obeyed a Lorentz force due to ambient magnetic field and applied opposing magnetic component that resulted in a measurable decrease of the ambient geomagnetic field. The magnitude of the magnetic field decrease depended on the distance between the HTS and magnetometer (S1 and S4 were a bit closer and resulted in over 200 nT magnetic field decrease, while S2 and S3 were a bit further and resulted in ~50 nT magnetic field decrease in Figure SM1).

Figure SM1: Four measurements (S1, S2, S3, S4) of temporal appearance of opposing magnetic field due to an onset of superconductivity in the high temperature superconductor (HTS) when to submerging into Liquid Nitrogen (77K). See Methods for details.

Following are details about the X-ray diffraction (XRD) analyses of the magnetic extract from the sub sample SF01, 43D (see methods).

Charles University in Prague – Faculty of Science, Institute of Geochemistry, Mineralogy and Mineral Resources, Albertov 6, 128 43 Prague 2


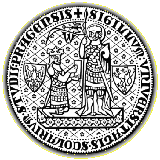


**analyst**: Viktor Golias, tel. 22195 1511, fax. 22195 1496, e-mail: wiki@ natur.cuni.cz

**equipment**: X´Pert Pro, PANalytical B.V., Almelo, the Netherlands

**software**: X´Pert HighScore 1.0d, PANalytical B.V., Almelo, the Netherlands

________________________________________________________________________________________________________________

## Measurement Conditions:

Sample Identification SF_MAG SEP CONC.xrdml

Measurement Date / Time 28/06/2021 17:16:06

Operator Administrator

Raw Data Origin XRD measurement (*.XRDML)

Scan Axis Gonio

Start Position [°2Th.] 3.0230

End Position [°2Th.] 59.9730

Step Size [°2Th.] 0.0500

Scan Step Time [s] 200.0250

Scan Type Continuous

PSD Mode Scanning

PSD Length [°2Th.] 2.12

Offset [°2Th.] 0.0000

Divergence Slit Type Automatic

Irradiated Length [mm] 10.00

Specimen Length [mm] 10.00

Measurement Temperature [°C] 25.00

Anode Material Cu

Generator Settings 40 kV, 30 mA

Goniometer Radius [mm] 240.00

Dist. Focus-Diverg. Slit [mm] 100.00

Incident Beam Monochromator No

Spinning Yes

## Peak List:

| Pos. [°2Th.] | d-spacing [Å] | Height [cts] | Rel. Int. [%] | FWHM [°2Th.] | Matched | Matched by |
| --- | --- | --- | --- | --- | --- | --- |
| 8.9089 | 9.92622 | 92.20 | 2.46 | 0.1476 | Yes | 99-000-0001 |
| 12.5690 | 7.04272 | 173.82 | 4.64 | 0.1476 | Yes | 00-034-0163 |
| 13.8261 | 6.40509 | 34.65 | 0.93 | 0.2952 | Yes | 01-084-0982; 00-022-0687 |
| 18.3710 | 4.82949 | 79.09 | 2.11 | 0.1476 | Yes | 01-088-0866 |
| 20.9240 | 4.24563 | 801.63 | 21.41 | 0.0984 | Yes | 01-086-1629; 00-022-0687 |
| 22.1214 | 4.01846 | 89.36 | 2.39 | 0.0984 | Yes | 01-084-0982 |
| 23.2036 | 3.83343 | 64.33 | 1.72 | 0.2460 | Yes | 01-084-0982; 00-022-0687; 99-000-0001; 00-034-0163 |
| 23.6308 | 3.76510 | 193.31 | 5.16 | 0.0984 | Yes | 01-084-0982 |
| 24.2202 | 3.67478 | 973.63 | 26.00 | 0.1968 | Yes | 01-079-0007; 01-084-0982; 00-012-0088; 00-022-0687 |
| 25.2949 | 3.52104 | 211.48 | 5.65 | 0.1476 | Yes | 01-084-0982; 00-034-0163 |
| 25.5930 | 3.48070 | 190.96 | 5.10 | 0.1476 | Yes | 01-084-0982; 00-022-0687; 99-000-0001 |
| 26.7129 | 3.33726 | 3744.09 | 100.00 | 0.1476 | Yes | 01-086-1629; 01-084-0982 |
| 27.5251 | 3.24061 | 1067.22 | 28.50 | 0.1476 | Yes | 00-022-0687 |
| 28.0003 | 3.18668 | 1100.76 | 29.40 | 0.1476 | Yes | 01-084-0982; 99-000-0001 |
| 30.1776 | 2.96155 | 466.59 | 12.46 | 0.1968 | Yes | 01-084-0982; 00-022-0687; 01-088-0866; 99-000-0001 |
| 30.8563 | 2.89793 | 118.16 | 3.16 | 0.1476 | Yes | 00-012-0088; 00-022-0687 |
| 33.2341 | 2.69583 | 3576.45 | 95.52 | 0.1968 | Yes | 01-079-0007; 00-012-0088 |
| 35.6799 | 2.51645 | 2776.20 | 74.15 | 0.3444 | Yes | 01-079-0007; 01-084-0982; 00-022-0687; 01-088-0866 |
| 36.6315 | 2.45323 | 309.63 | 8.27 | 0.1968 | Yes | 01-086-1629; 01-084-0982; 99-000-0001 |
| 37.1280 | 2.42156 | 122.90 | 3.28 | 0.1476 | Yes | 01-084-0982; 00-012-0088; 00-022-0687; 01-088-0866; 99-000-0001 |
| 39.5446 | 2.27897 | 229.30 | 6.12 | 0.1968 | Yes | 01-086-1629; 01-084-0982 |
| 40.3777 | 2.23385 | 139.94 | 3.74 | 0.1476 | Yes | 01-086-1629; 01-084-0982; 00-022-0687; 99-000-0001 |
| 40.9424 | 2.20434 | 758.48 | 20.26 | 0.1968 | Yes | 01-079-0007; 00-012-0088; 99-000-0001 |
| 41.8748 | 2.15739 | 110.69 | 2.96 | 0.1968 | Yes | 01-084-0982; 00-022-0687 |
| 42.5452 | 2.12493 | 217.19 | 5.80 | 0.1476 | Yes | 01-086-1629; 01-084-0982; 99-000-0001; 00-034-0163 |
| 43.1432 | 2.09685 | 287.13 | 7.67 | 0.1968 | Yes | 01-084-0982; 00-022-0687; 01-088-0866; 99-000-0001 |
| 45.5464 | 1.99165 | 81.22 | 2.17 | 0.1476 | Yes | 01-084-0982; 00-022-0687; 99-000-0001 |
| 45.8793 | 1.97797 | 131.74 | 3.52 | 0.1476 | Yes | 01-086-1629; 01-084-0982; 00-022-0687; 99-000-0001 |
| 47.2160 | 1.92505 | 34.12 | 0.91 | 0.2952 | Yes | 01-084-0982; 00-022-0687; 01-088-0866; 99-000-0001; 00-034-0163 |
| 48.2282 | 1.88698 | 35.81 | 0.96 | 0.2952 | Yes | 01-084-0982; 00-022-0687; 99-000-0001 |
| 49.5447 | 1.83988 | 937.25 | 25.03 | 0.2460 | Yes | 01-079-0007; 01-084-0982; 99-000-0001 |
| 50.2306 | 1.81636 | 508.35 | 13.58 | 0.1968 | Yes | 01-086-1629; 01-084-0982; 00-012-0088; 99-000-0001 |
| 50.6305 | 1.80295 | 94.82 | 2.53 | 0.1476 | Yes | 01-086-1629; 01-084-0982 |
| 53.5339 | 1.71181 | 116.29 | 3.11 | 0.1968 | Yes | 01-084-0982; 01-088-0866 |
| 54.1525 | 1.69371 | 1109.75 | 29.64 | 0.2952 | Yes | 01-079-0007; 01-084-0982; 99-000-0001; 00-034-0163 |
| 54.9936 | 1.66978 | 103.98 | 2.78 | 0.2460 | Yes | 01-086-1629; 01-084-0982; 99-000-0001 |
| 57.0309 | 1.61488 | 319.71 | 8.54 | 0.1968 | Yes | 01-084-0982; 01-088-0866; 99-000-0001 |
| 57.6547 | 1.59756 | 258.91 | 6.92 | 0.2400 | Yes | 01-079-0007; 01-084-0982; 99-000-0001; 00-034-0163 |

## Analyze View:

## Pattern List:

| Ref. Code | Compound Name | Score | Total Lines | Scale Factor | SemiQuant [%] |
| --- | --- | --- | --- | --- | --- |
| 01-086-1629 | Quartz low | 48 | 12 | 0.987 | 30 |
| 01-079-0007 | Hematite | 50 | 11 | 0.790 | 23 |
| 01-084-0982 | Albite low | 40 | 124 | 0.187 | 27 |
| 00-012-0088 | Ankerite | 15 | 13 | 0.038 | 1 |
| 00-022-0687 | Microcline, ordered | 37 | 56 | 0.116 | 4 |
| 01-088-0866 | Magnetite (Cr-bearing) | 29 | 8 | 0.428 | 8 |
| 99-000-0001 | Muscovite | 21 | 92 | 0.063 | 3 |
| 00-034-0163 | Amesite-2\ITH\RG#2 | 20 | 22 | 0.045 | 4 |

References:

1 Kletetschka, G. & Wieczorek, M. A. Fundamental Relations of Mineral Specific Magnetic Carriers for Paleointensity Determination. *Physics of the Earth and Planetary Interiors* **272**, 44-49, doi:<https://doi.org/10.1016/j.pepi.2017.09.008> (2017).

2 Néel, L. Théorie du traînage magnétique des ferromagnétiques en grains fins avec applications aux terres cuites. *Annales de Geophysique* **5**, 99-136 (1949).
