# Supplementary material for: Plasma shielding removes prior magnetization record from impacted rocks near Santa Fe, New Mexico
Source: Sci Rep. 2021 Nov 17;11:22466. doi: 10.1038/s41598-021-01451-8 (PMC8599688; doi:10.1038/s41598-021-01451-8)
Supplement: Supplementary file 2 — Supplementary Legends. [file 41598_2021_1451_MOESM2_ESM.docx]

Legends for supplementary figures and tables listed in supplementary excel files:

Figure S1: Stepwise demagnetization (3, 5, 7, 10, 15, 20, 25, 30, 40, 50 A/m) of Natural Remanent Magnetization (NRM) of sample 11A is shown in A in three types of plots. Upper left shows how direction of NRM changed during the demagnetization (NRM + 10 steps, N=10). Black circle points down, white points up. Cross is the NRM direction prior to demagnetization steps. Upper right is projection of the stepwise demagnetized remanence vector onto horizontal (+X+Y, gray circle) and vertical (-Z+Y, white circle) plane. Lower left is the stepwise demagnetized magnitude of the remanence vector normalized by stated maximum. Stepwise demagnetization of Saturation Isothermal Remanent Magnetization (SIRM) of sample 11A (labelled 11A_PULS) is shown in B using the same types of plots as in A. Data used for plotting these figures are listed under the figures.

Figure S2: Stepwise demagnetization (3, 5, 7, 10, 15, 20, 25, 30, 40, 50 A/m) of Natural Remanent Magnetization (NRM) of sample 11B is shown in A in three types of plots. Upper left shows how direction of NRM changed during the demagnetization (NRM + 10 steps, N=10). Black circle points down, white points up. Cross is the NRM direction prior to demagnetization steps. Upper right is projection of the stepwise demagnetized remanence vector onto horizontal (+X+Y, gray circle) and vertical (-Z+Y, white circle) plane. Lower left is the stepwise demagnetized magnitude of the remanence vector normalized by stated maximum. Stepwise demagnetization of Saturation Isothermal Remanent Magnetization (SIRM) of sample 11B (labelled 11B_PULS) is shown in B using the same types of plots as in A. Data used for plotting these figures are listed under the figures.

Figure S3: Stepwise demagnetization (3, 5, 7, 10, 15, 20, 25, 30, 40, 50 A/m) of Natural Remanent Magnetization (NRM) of sample 11C is shown in A in three types of plots. Upper left shows how direction of NRM changed during the demagnetization (NRM + 10 steps, N=10). Black circle points down, white points up. Cross is the NRM direction prior to demagnetization steps. Upper right is projection of the stepwise demagnetized remanence vector onto horizontal (+X+Y, gray circle) and vertical (-Z+Y, white circle) plane. Lower left is the stepwise demagnetized magnitude of the remanence vector normalized by stated maximum. Stepwise demagnetization of Saturation Isothermal Remanent Magnetization (SIRM) of sample 11C (labelled 11C_PULS) is shown in B using the same types of plots as in A. Data used for plotting these figures are listed under the figures.

Figure S4: Stepwise demagnetization (3, 5, 7, 10, 15, 20, 25, 30, 40, 50 A/m) of Natural Remanent Magnetization (NRM) of sample 12A is shown in A in three types of plots. Upper left shows how direction of NRM changed during the demagnetization (NRM + 10 steps, N=10). Black circle points down, white points up. Cross is the NRM direction prior to demagnetization steps. Upper right is projection of the stepwise demagnetized remanence vector onto horizontal (+X+Y, gray circle) and vertical (-Z+Y, white circle) plane. Lower left is the stepwise demagnetized magnitude of the remanence vector normalized by stated maximum. Stepwise demagnetization of Saturation Isothermal Remanent Magnetization (SIRM) of sample 12A (labelled 12A_PULS) is shown in B using the same types of plots as in A. Data used for plotting these figures are listed under the figures.

Figure S5: Stepwise demagnetization (3, 5, 7, 10, 15, 20, 25, 30, 40, 50 A/m) of Natural Remanent Magnetization (NRM) of sample 12D is shown in A in three types of plots. Upper left shows how direction of NRM changed during the demagnetization (NRM + 10 steps, N=10). Black circle points down, white points up. Cross is the NRM direction prior to demagnetization steps. Upper right is projection of the stepwise demagnetized remanence vector onto horizontal (+X+Y, gray circle) and vertical (-Z+Y, white circle) plane. Lower left is the stepwise demagnetized magnitude of the remanence vector normalized by stated maximum. Stepwise demagnetization of Saturation Isothermal Remanent Magnetization (SIRM) of sample 12D (labelled 12D_PULS) is shown in B using the same types of plots as in A. Data used for plotting these figures are listed under the figures.

Figure S6: Stepwise demagnetization (3, 5, 7, 10, 15, 20, 25, 30, 40, 50 A/m) of Natural Remanent Magnetization (NRM) of sample 14C is shown in A in three types of plots. Upper left shows how direction of NRM changed during the demagnetization (NRM + 10 steps, N=10). Black circle points down, white points up. Cross is the NRM direction prior to demagnetization steps. Upper right is projection of the stepwise demagnetized remanence vector onto horizontal (+X+Y, gray circle) and vertical (-Z+Y, white circle) plane. Lower left is the stepwise demagnetized magnitude of the remanence vector normalized by stated maximum. Stepwise demagnetization of Saturation Isothermal Remanent Magnetization (SIRM) of sample 14C (labelled 14C_PULS) is shown in B using the same types of plots as in A. Data used for plotting these figures are listed under the figures.

Figure S7: Stepwise demagnetization (3, 5, 7, 10, 15, 20, 25, 30, 40, 50 A/m) of Natural Remanent Magnetization (NRM) of sample 14D is shown in A in three types of plots. Upper left shows how direction of NRM changed during the demagnetization (NRM + 10 steps, N=10). Black circle points down, white points up. Cross is the NRM direction prior to demagnetization steps. Upper right is projection of the stepwise demagnetized remanence vector onto horizontal (+X+Y, gray circle) and vertical (-Z+Y, white circle) plane. Lower left is the stepwise demagnetized magnitude of the remanence vector normalized by stated maximum. Stepwise demagnetization of Saturation Isothermal Remanent Magnetization (SIRM) of sample 14D (labelled 14D_PULS) is shown in B using the same types of plots as in A. Data used for plotting these figures are listed under the figures.

Figure S8: Stepwise demagnetization (3, 5, 7, 10, 15, 20, 25, 30, 40, 50 A/m) of Natural Remanent Magnetization (NRM) of sample 15B is shown in A in three types of plots. Upper left shows how direction of NRM changed during the demagnetization (NRM + 10 steps, N=10). Black circle points down, white points up. Cross is the NRM direction prior to demagnetization steps. Upper right is projection of the stepwise demagnetized remanence vector onto horizontal (+X+Y, gray circle) and vertical (-Z+Y, white circle) plane. Lower left is the stepwise demagnetized magnitude of the remanence vector normalized by stated maximum. Stepwise demagnetization of Saturation Isothermal Remanent Magnetization (SIRM) of sample 15B (labelled 15B_PULS) is shown in B using the same types of plots as in A. Data used for plotting these figures are listed under the figures.

Figure S9: Stepwise demagnetization (3, 5, 7, 10, 15, 20, 25, 30, 40, 50 A/m) of Natural Remanent Magnetization (NRM) of sample 15C is shown in A in three types of plots. Upper left shows how direction of NRM changed during the demagnetization (NRM + 10 steps, N=10). Black circle points down, white points up. Cross is the NRM direction prior to demagnetization steps. Upper right is projection of the stepwise demagnetized remanence vector onto horizontal (+X+Y, gray circle) and vertical (-Z+Y, white circle) plane. Lower left is the stepwise demagnetized magnitude of the remanence vector normalized by stated maximum. Stepwise demagnetization of Saturation Isothermal Remanent Magnetization (SIRM) of sample 15C (labelled 15C_PULS) is shown in B using the same types of plots as in A. Data used for plotting these figures are listed under the figures.

Figure S10: Stepwise demagnetization (3, 5, 7, 10, 15, 20, 25, 30, 40, 50 A/m) of Natural Remanent Magnetization (NRM) of sample 15D is shown in A in three types of plots. Upper left shows how direction of NRM changed during the demagnetization (NRM + 10 steps, N=10). Black circle points down, white points up. Cross is the NRM direction prior to demagnetization steps. Upper right is projection of the stepwise demagnetized remanence vector onto horizontal (+X+Y, gray circle) and vertical (-Z+Y, white circle) plane. Lower left is the stepwise demagnetized magnitude of the remanence vector normalized by stated maximum. Stepwise demagnetization of Saturation Isothermal Remanent Magnetization (SIRM) of sample 15D (labelled 15D_PULS) is shown in B using the same types of plots as in A. Data used for plotting these figures are listed under the figures.

Figure S11: Stepwise demagnetization (3, 5, 7, 10, 15, 20, 25, 30, 40, 50 A/m) of Natural Remanent Magnetization (NRM) of sample 21D is shown in A in three types of plots. Upper left shows how direction of NRM changed during the demagnetization (NRM + 10 steps, N=10). Black circle points down, white points up. Cross is the NRM direction prior to demagnetization steps. Upper right is projection of the stepwise demagnetized remanence vector onto horizontal (+X+Y, gray circle) and vertical (-Z+Y, white circle) plane. Lower left is the stepwise demagnetized magnitude of the remanence vector normalized by stated maximum. Stepwise demagnetization of Saturation Isothermal Remanent Magnetization (SIRM) of sample 21D (labelled 21D_PULS) is shown in B using the same types of plots as in A. Data used for plotting these figures are listed under the figures.

Figure S12: Stepwise demagnetization (3, 5, 7, 10, 15, 20, 25, 30, 40, 50 A/m) of Natural Remanent Magnetization (NRM) of sample 22D is shown in A in three types of plots. Upper left shows how direction of NRM changed during the demagnetization (NRM + 10 steps, N=10). Black circle points down, white points up. Cross is the NRM direction prior to demagnetization steps. Upper right is projection of the stepwise demagnetized remanence vector onto horizontal (+X+Y, gray circle) and vertical (-Z+Y, white circle) plane. Lower left is the stepwise demagnetized magnitude of the remanence vector normalized by stated maximum. Stepwise demagnetization of Saturation Isothermal Remanent Magnetization (SIRM) of sample 22D (labelled 22D_PULS) is shown in B using the same types of plots as in A. Data used for plotting these figures are listed under the figures.

Figure S13: Stepwise demagnetization (3, 5, 7, 10, 15, 20, 25, 30, 40, 50 A/m) of Natural Remanent Magnetization (NRM) of sample 24D is shown in A in three types of plots. Upper left shows how direction of NRM changed during the demagnetization (NRM + 10 steps, N=10). Black circle points down, white points up. Cross is the NRM direction prior to demagnetization steps. Upper right is projection of the stepwise demagnetized remanence vector onto horizontal (+X+Y, gray circle) and vertical (-Z+Y, white circle) plane. Lower left is the stepwise demagnetized magnitude of the remanence vector normalized by stated maximum. Stepwise demagnetization of Saturation Isothermal Remanent Magnetization (SIRM) of sample 24D (labelled 24D_PULS) is shown in B using the same types of plots as in A. Data used for plotting these figures are listed under the figures.

Figure S14: Stepwise demagnetization (3, 5, 7, 10, 15, 20, 25, 30, 40, 50 A/m) of Natural Remanent Magnetization (NRM) of sample 25A is shown in A in three types of plots. Upper left shows how direction of NRM changed during the demagnetization (NRM + 10 steps, N=10). Black circle points down, white points up. Cross is the NRM direction prior to demagnetization steps. Upper right is projection of the stepwise demagnetized remanence vector onto horizontal (+X+Y, gray circle) and vertical (-Z+Y, white circle) plane. Lower left is the stepwise demagnetized magnitude of the remanence vector normalized by stated maximum. Stepwise demagnetization of Saturation Isothermal Remanent Magnetization (SIRM) of sample 25A (labelled 25A_PULS) is shown in B using the same types of plots as in A. Data used for plotting these figures are listed under the figures.

Figure S15: Stepwise demagnetization (3, 5, 7, 10, 15, 20, 25, 30, 40, 50 A/m) of Natural Remanent Magnetization (NRM) of sample 25B is shown in A in three types of plots. Upper left shows how direction of NRM changed during the demagnetization (NRM + 10 steps, N=10). Black circle points down, white points up. Cross is the NRM direction prior to demagnetization steps. Upper right is projection of the stepwise demagnetized remanence vector onto horizontal (+X+Y, gray circle) and vertical (-Z+Y, white circle) plane. Lower left is the stepwise demagnetized magnitude of the remanence vector normalized by stated maximum. Stepwise demagnetization of Saturation Isothermal Remanent Magnetization (SIRM) of sample 25B (labelled 25B_PULS) is shown in B using the same types of plots as in A. Data used for plotting these figures are listed under the figures.

Figure S16: Stepwise demagnetization (3, 5, 7, 10, 15, 20, 25, 30, 40, 50 A/m) of Natural Remanent Magnetization (NRM) of sample 31D is shown in A in three types of plots. Upper left shows how direction of NRM changed during the demagnetization (NRM + 10 steps, N=10). Black circle points down, white points up. Cross is the NRM direction prior to demagnetization steps. Upper right is projection of the stepwise demagnetized remanence vector onto horizontal (+X+Y, gray circle) and vertical (-Z+Y, white circle) plane. Lower left is the stepwise demagnetized magnitude of the remanence vector normalized by stated maximum. Stepwise demagnetization of Saturation Isothermal Remanent Magnetization (SIRM) of sample 31D (labelled 31D_PULS) is shown in B using the same types of plots as in A. Data used for plotting these figures are listed under the figures.

Figure S17: Stepwise demagnetization (3, 5, 7, 10, 15, 20, 25, 30, 40, 50 A/m) of Natural Remanent Magnetization (NRM) of sample 34A is shown in A in three types of plots. Upper left shows how direction of NRM changed during the demagnetization (NRM + 10 steps, N=10). Black circle points down, white points up. Cross is the NRM direction prior to demagnetization steps. Upper right is projection of the stepwise demagnetized remanence vector onto horizontal (+X+Y, gray circle) and vertical (-Z+Y, white circle) plane. Lower left is the stepwise demagnetized magnitude of the remanence vector normalized by stated maximum. Stepwise demagnetization of Saturation Isothermal Remanent Magnetization (SIRM) of sample 34A (labelled 34A_PULS) is shown in B using the same types of plots as in A. Data used for plotting these figures are listed under the figures.

Figure S18: Stepwise demagnetization (3, 5, 7, 10, 15, 20, 25, 30, 40, 50 A/m) of Natural Remanent Magnetization (NRM) of sample 34B is shown in A in three types of plots. Upper left shows how direction of NRM changed during the demagnetization (NRM + 10 steps, N=10). Black circle points down, white points up. Cross is the NRM direction prior to demagnetization steps. Upper right is projection of the stepwise demagnetized remanence vector onto horizontal (+X+Y, gray circle) and vertical (-Z+Y, white circle) plane. Lower left is the stepwise demagnetized magnitude of the remanence vector normalized by stated maximum. Stepwise demagnetization of Saturation Isothermal Remanent Magnetization (SIRM) of sample 34B (labelled 34B_PULS) is shown in B using the same types of plots as in A. Data used for plotting these figures are listed under the figures.

Figure S19: Stepwise demagnetization (3, 5, 7, 10, 15, 20, 25, 30, 40, 50 A/m) of Natural Remanent Magnetization (NRM) of sample 35A is shown in A in three types of plots. Upper left shows how direction of NRM changed during the demagnetization (NRM + 10 steps, N=10). Black circle points down, white points up. Cross is the NRM direction prior to demagnetization steps. Upper right is projection of the stepwise demagnetized remanence vector onto horizontal (+X+Y, gray circle) and vertical (-Z+Y, white circle) plane. Lower left is the stepwise demagnetized magnitude of the remanence vector normalized by stated maximum. Stepwise demagnetization of Saturation Isothermal Remanent Magnetization (SIRM) of sample 35A (labelled 35A_PULS) is shown in B using the same types of plots as in A. Data used for plotting these figures are listed under the figures.

Figure S20: Stepwise demagnetization (3, 5, 7, 10, 15, 20, 25, 30, 40, 50 A/m) of Natural Remanent Magnetization (NRM) of sample 35B is shown in A in three types of plots. Upper left shows how direction of NRM changed during the demagnetization (NRM + 10 steps, N=10). Black circle points down, white points up. Cross is the NRM direction prior to demagnetization steps. Upper right is projection of the stepwise demagnetized remanence vector onto horizontal (+X+Y, gray circle) and vertical (-Z+Y, white circle) plane. Lower left is the stepwise demagnetized magnitude of the remanence vector normalized by stated maximum. Stepwise demagnetization of Saturation Isothermal Remanent Magnetization (SIRM) of sample 35B (labelled 35B_PULS) is shown in B using the same types of plots as in A. Data used for plotting these figures are listed under the figures.

Figure S21: Stepwise demagnetization (3, 5, 7, 10, 15, 20, 25, 30, 40, 50 A/m) of Natural Remanent Magnetization (NRM) of sample 35C is shown in A in three types of plots. Upper left shows how direction of NRM changed during the demagnetization (NRM + 10 steps, N=10). Black circle points down, white points up. Cross is the NRM direction prior to demagnetization steps. Upper right is projection of the stepwise demagnetized remanence vector onto horizontal (+X+Y, gray circle) and vertical (-Z+Y, white circle) plane. Lower left is the stepwise demagnetized magnitude of the remanence vector normalized by stated maximum. Stepwise demagnetization of Saturation Isothermal Remanent Magnetization (SIRM) of sample 35C (labelled 35C_PULS) is shown in B using the same types of plots as in A. Data used for plotting these figures are listed under the figures.

Figure S22: Stepwise demagnetization (3, 5, 7, 10, 15, 20, 25, 30, 40, 50 A/m) of Natural Remanent Magnetization (NRM) of sample 41B is shown in A in three types of plots. Upper left shows how direction of NRM changed during the demagnetization (NRM + 10 steps, N=10). Black circle points down, white points up. Cross is the NRM direction prior to demagnetization steps. Upper right is projection of the stepwise demagnetized remanence vector onto horizontal (+X+Y, gray circle) and vertical (-Z+Y, white circle) plane. Lower left is the stepwise demagnetized magnitude of the remanence vector normalized by stated maximum. Stepwise demagnetization of Saturation Isothermal Remanent Magnetization (SIRM) of sample 41B (labelled 41B_PULS) is shown in B using the same types of plots as in A. Data used for plotting these figures are listed under the figures.

Figure S23: Stepwise demagnetization (3, 5, 7, 10, 15, 20, 25, 30, 40, 50 A/m) of Natural Remanent Magnetization (NRM) of sample 41C is shown in A in three types of plots. Upper left shows how direction of NRM changed during the demagnetization (NRM + 10 steps, N=10). Black circle points down, white points up. Cross is the NRM direction prior to demagnetization steps. Upper right is projection of the stepwise demagnetized remanence vector onto horizontal (+X+Y, gray circle) and vertical (-Z+Y, white circle) plane. Lower left is the stepwise demagnetized magnitude of the remanence vector normalized by stated maximum. Stepwise demagnetization of Saturation Isothermal Remanent Magnetization (SIRM) of sample 41C (labelled 41C_PULS) is shown in B using the same types of plots as in A. Data used for plotting these figures are listed under the figures.

Figure S24: Stepwise demagnetization (3, 5, 7, 10, 15, 20, 25, 30, 40, 50 A/m) of Natural Remanent Magnetization (NRM) of sample 41D is shown in A in three types of plots. Upper left shows how direction of NRM changed during the demagnetization (NRM + 10 steps, N=10). Black circle points down, white points up. Cross is the NRM direction prior to demagnetization steps. Upper right is projection of the stepwise demagnetized remanence vector onto horizontal (+X+Y, gray circle) and vertical (-Z+Y, white circle) plane. Lower left is the stepwise demagnetized magnitude of the remanence vector normalized by stated maximum. Stepwise demagnetization of Saturation Isothermal Remanent Magnetization (SIRM) of sample 41D (labelled 41D_PULS) is shown in B using the same types of plots as in A. Data used for plotting these figures are listed under the figures.

Figure S25: Stepwise demagnetization (3, 5, 7, 10, 15, 20, 25, 30, 40, 50 A/m) of Natural Remanent Magnetization (NRM) of sample 43D is shown in A in three types of plots. Upper left shows how direction of NRM changed during the demagnetization (NRM + 10 steps, N=10). Black circle points down, white points up. Cross is the NRM direction prior to demagnetization steps. Upper right is projection of the stepwise demagnetized remanence vector onto horizontal (+X+Y, gray circle) and vertical (-Z+Y, white circle) plane. Lower left is the stepwise demagnetized magnitude of the remanence vector normalized by stated maximum. Stepwise demagnetization of Saturation Isothermal Remanent Magnetization (SIRM) of sample 43D (labelled 43D_PULS) is shown in B using the same types of plots as in A. Data used for plotting these figures are listed under the figures.

Figure S26: Stepwise demagnetization (3, 5, 7, 10, 15, 20, 25, 30, 40, 50 A/m) of Natural Remanent Magnetization (NRM) of sample 51C is shown in A in three types of plots. Upper left shows how direction of NRM changed during the demagnetization (NRM + 10 steps, N=10). Black circle points down, white points up. Cross is the NRM direction prior to demagnetization steps. Upper right is projection of the stepwise demagnetized remanence vector onto horizontal (+X+Y, gray circle) and vertical (-Z+Y, white circle) plane. Lower left is the stepwise demagnetized magnitude of the remanence vector normalized by stated maximum. Stepwise demagnetization of Saturation Isothermal Remanent Magnetization (SIRM) of sample 51C (labelled 51C_PULS) is shown in B using the same types of plots as in A. Data used for plotting these figures are listed under the figures.

Figure S27: Stepwise demagnetization (3, 5, 7, 10, 15, 20, 25, 30, 40, 50 A/m) of Natural Remanent Magnetization (NRM) of sample 53D is shown in A in three types of plots. Upper left shows how direction of NRM changed during the demagnetization (NRM + 10 steps, N=10). Black circle points down, white points up. Cross is the NRM direction prior to demagnetization steps. Upper right is projection of the stepwise demagnetized remanence vector onto horizontal (+X+Y, gray circle) and vertical (-Z+Y, white circle) plane. Lower left is the stepwise demagnetized magnitude of the remanence vector normalized by stated maximum. Stepwise demagnetization of Saturation Isothermal Remanent Magnetization (SIRM) of sample 53D (labelled 53D_PULS) is shown in B using the same types of plots as in A. Data used for plotting these figures are listed under the figures.

Figure S28: Stepwise demagnetization (3, 5, 7, 10, 15, 20, 25, 30, 40, 50 A/m) of Natural Remanent Magnetization (NRM) of sample 29A is shown in A in three types of plots. Upper left shows how direction of NRM changed during the demagnetization (NRM + 10 steps, N=10). Black circle points down, white points up. Cross is the NRM direction prior to demagnetization steps. Upper right is projection of the stepwise demagnetized remanence vector onto horizontal (+X+Y, gray circle) and vertical (-Z+Y, white circle) plane. Lower left is the stepwise demagnetized magnitude of the remanence vector normalized by stated maximum. Stepwise demagnetization of Saturation Isothermal Remanent Magnetization (SIRM) of sample 29A (labelled 29A_PULS) is shown in B using the same types of plots as in A. Data used for plotting these figures are listed in the lower right corners.

Figure S29: Stepwise demagnetization (3, 5, 7, 10, 15, 20, 25, 30, 40, 50 A/m) of Natural Remanent Magnetization (NRM) of sample 29B is shown in A in three types of plots. Upper left shows how direction of NRM changed during the demagnetization (NRM + 10 steps, N=10). Black circle points down, white points up. Cross is the NRM direction prior to demagnetization steps. Upper right is projection of the stepwise demagnetized remanence vector onto horizontal (+X+Y, gray circle) and vertical (-Z+Y, white circle) plane. Lower left is the stepwise demagnetized magnitude of the remanence vector normalized by stated maximum. Stepwise demagnetization of Saturation Isothermal Remanent Magnetization (SIRM) of sample 29B (labelled 29B_PULS) is shown in B using the same types of plots as in A. Data used for plotting these figures are listed in the lower right corners.

Figure S30: Stepwise demagnetization (3, 5, 7, 10, 15, 20, 25, 30, 40, 50 A/m) of Natural Remanent Magnetization (NRM) of sample 31A is shown in A in three types of plots. Upper left shows how direction of NRM changed during the demagnetization (NRM + 10 steps, N=10). Black circle points down, white points up. Cross is the NRM direction prior to demagnetization steps. Upper right is projection of the stepwise demagnetized remanence vector onto horizontal (+X+Y, gray circle) and vertical (-Z+Y, white circle) plane. Lower left is the stepwise demagnetized magnitude of the remanence vector normalized by stated maximum. Stepwise demagnetization of Saturation Isothermal Remanent Magnetization (SIRM) of sample 31A (labelled 31A_PULS) is shown in B using the same types of plots as in A. Data used for plotting these figures are listed in the lower right corners.

Figure S31: Stepwise demagnetization (3, 5, 7, 10, 15, 20, 25, 30, 40, 50 A/m) of Natural Remanent Magnetization (NRM) of sample 31B is shown in A in three types of plots. Upper left shows how direction of NRM changed during the demagnetization (NRM + 10 steps, N=10). Black circle points down, white points up. Cross is the NRM direction prior to demagnetization steps. Upper right is projection of the stepwise demagnetized remanence vector onto horizontal (+X+Y, gray circle) and vertical (-Z+Y, white circle) plane. Lower left is the stepwise demagnetized magnitude of the remanence vector normalized by stated maximum. Stepwise demagnetization of Saturation Isothermal Remanent Magnetization (SIRM) of sample 31B (labelled 31B_PULS) is shown in B using the same types of plots as in A. Data used for plotting these figures are listed in the lower right corners.

Figure S32: Four measurements (S1, S2, S3, S4) of temporal appearance of opposing magnetic field due to an onset of superconductivity in the high temperature superconductor (HTS) when to submerging into Liquid Nitrogen (77K). See Methods for details.

Table S1: Magnetic data for sample 11A, when demagnetized by alternating magnetic field. Column A: Marked as “ID”. Identification name of the sample. When the name is followed by letters “PULS”, the sample’s magnetic state was reset be exposing to 1T pulse magnetic field and demagnetized in the same set of steps as prior to magnetic field exposure. Column B: Marked as: ”AF[mT]”. It has alphanumerical label, where AD stands for “alternating demagnetization” and is followed by number indicating a peak of alternating magnetic field in units of milli Tesla [mT]. Columns C, D, E: Marked as “x”, “y”, “z”. Detection of the three perpendicular magnetic components characterizing the magnetic strength of the sample. To get the proper units of this measurements, the number needs to be multiplied by power of 10 listed in column F. The magnitude of the magnetic vector is listed in column R (M [A/m]). Column S shows normalized magnetic vector amplitudes to its initial value listed before the onset of demagnetization. Column T shows a level of demagnetization by alternating magnetic field in [mT]. Column U shows magnetic efficiency obtained by ratio of non-saturated and saturated values demagnetized to specific level of alternating magnetic field, labelled as “REM”. Column V shows estimating paleofield assuming that the magnetic carrier in this sample is magnetite (used multiplication factor of 2800^1^).

Table S2: Magnetic data for sample 11B, when demagnetized by alternating magnetic field. Column A: Marked as “ID”. Identification name of the sample. When the name is followed by letters “PULS”, the sample’s magnetic state was reset be exposing to 1T pulse magnetic field and demagnetized in the same set of steps as prior to magnetic field exposure. Column B: Marked as: ”AF[mT]”. It has alphanumerical label, where AD stands for “alternating demagnetization” and is followed by number indicating a peak of alternating magnetic field in units of milli Tesla [mT]. Columns C, D, E: Marked as “x”, “y”, “z”. Detection of the three perpendicular magnetic components characterizing the magnetic strength of the sample. To get the proper units of this measurements, the number needs to be multiplied by power of 10 listed in column F. The magnitude of the magnetic vector is listed in column R (M [A/m]). Column S shows normalized magnetic vector amplitudes to its initial value listed before the onset of demagnetization. Column T shows a level of demagnetization by alternating magnetic field in [mT]. Column U shows magnetic efficiency obtained by ratio of non-saturated and saturated values demagnetized to specific level of alternating magnetic field, labelled as “REM”. Column V shows estimating paleofield assuming that the magnetic carrier in this sample is magnetite (used multiplication factor of 2800^1^).

Table S3: Magnetic data for sample 11C, when demagnetized by alternating magnetic field. Column A: Marked as “ID”. Identification name of the sample. When the name is followed by letters “PULS”, the sample’s magnetic state was reset be exposing to 1T pulse magnetic field and demagnetized in the same set of steps as prior to magnetic field exposure. Column B: Marked as: ”AF[mT]”. It has alphanumerical label, where AD stands for “alternating demagnetization” and is followed by number indicating a peak of alternating magnetic field in units of milli Tesla [mT]. Columns C, D, E: Marked as “x”, “y”, “z”. Detection of the three perpendicular magnetic components characterizing the magnetic strength of the sample. To get the proper units of this measurements, the number needs to be multiplied by power of 10 listed in column F. The magnitude of the magnetic vector is listed in column R (M [A/m]). Column S shows normalized magnetic vector amplitudes to its initial value listed before the onset of demagnetization. Column T shows a level of demagnetization by alternating magnetic field in [mT]. Column U shows magnetic efficiency obtained by ratio of non-saturated and saturated values demagnetized to specific level of alternating magnetic field, labelled as “REM”. Column V shows estimating paleofield assuming that the magnetic carrier in this sample is magnetite (used multiplication factor of 2800^1^).

Table S4: Magnetic data for sample 12A, when demagnetized by alternating magnetic field. Column A: Marked as “ID”. Identification name of the sample. When the name is followed by letters “PULS”, the sample’s magnetic state was reset be exposing to 1T pulse magnetic field and demagnetized in the same set of steps as prior to magnetic field exposure. Column B: Marked as: ”AF[mT]”. It has alphanumerical label, where AD stands for “alternating demagnetization” and is followed by number indicating a peak of alternating magnetic field in units of milli Tesla [mT]. Columns C, D, E: Marked as “x”, “y”, “z”. Detection of the three perpendicular magnetic components characterizing the magnetic strength of the sample. To get the proper units of this measurements, the number needs to be multiplied by power of 10 listed in column F. The magnitude of the magnetic vector is listed in column R (M [A/m]). Column S shows normalized magnetic vector amplitudes to its initial value listed before the onset of demagnetization. Column T shows a level of demagnetization by alternating magnetic field in [mT]. Column U shows magnetic efficiency obtained by ratio of non-saturated and saturated values demagnetized to specific level of alternating magnetic field, labelled as “REM”. Column V shows estimating paleofield assuming that the magnetic carrier in this sample is magnetite (used multiplication factor of 2800^1^).

Table S5: Magnetic data for sample 12D, when demagnetized by alternating magnetic field. Column A: Marked as “ID”. Identification name of the sample. When the name is followed by letters “PULS”, the sample’s magnetic state was reset be exposing to 1T pulse magnetic field and demagnetized in the same set of steps as prior to magnetic field exposure. Column B: Marked as: ”AF[mT]”. It has alphanumerical label, where AD stands for “alternating demagnetization” and is followed by number indicating a peak of alternating magnetic field in units of milli Tesla [mT]. Columns C, D, E: Marked as “x”, “y”, “z”. Detection of the three perpendicular magnetic components characterizing the magnetic strength of the sample. To get the proper units of this measurements, the number needs to be multiplied by power of 10 listed in column F. The magnitude of the magnetic vector is listed in column R (M [A/m]). Column S shows normalized magnetic vector amplitudes to its initial value listed before the onset of demagnetization. Column T shows a level of demagnetization by alternating magnetic field in [mT]. Column U shows magnetic efficiency obtained by ratio of non-saturated and saturated values demagnetized to specific level of alternating magnetic field, labelled as “REM”. Column V shows estimating paleofield assuming that the magnetic carrier in this sample is magnetite (used multiplication factor of 2800^1^).

Table S6: Magnetic data for sample 14C, when demagnetized by alternating magnetic field. Column A: Marked as “ID”. Identification name of the sample. When the name is followed by letters “PULS”, the sample’s magnetic state was reset be exposing to 1T pulse magnetic field and demagnetized in the same set of steps as prior to magnetic field exposure. Column B: Marked as: ”AF[mT]”. It has alphanumerical label, where AD stands for “alternating demagnetization” and is followed by number indicating a peak of alternating magnetic field in units of milli Tesla [mT]. Columns C, D, E: Marked as “x”, “y”, “z”. Detection of the three perpendicular magnetic components characterizing the magnetic strength of the sample. To get the proper units of this measurements, the number needs to be multiplied by power of 10 listed in column F. The magnitude of the magnetic vector is listed in column R (M [A/m]). Column S shows normalized magnetic vector amplitudes to its initial value listed before the onset of demagnetization. Column T shows a level of demagnetization by alternating magnetic field in [mT]. Column U shows magnetic efficiency obtained by ratio of non-saturated and saturated values demagnetized to specific level of alternating magnetic field, labelled as “REM”. Column V shows estimating paleofield assuming that the magnetic carrier in this sample is magnetite (used multiplication factor of 2800^1^).

Table S7: Magnetic data for sample 14D, when demagnetized by alternating magnetic field. Column A: Marked as “ID”. Identification name of the sample. When the name is followed by letters “PULS”, the sample’s magnetic state was reset be exposing to 1T pulse magnetic field and demagnetized in the same set of steps as prior to magnetic field exposure. Column B: Marked as: ”AF[mT]”. It has alphanumerical label, where AD stands for “alternating demagnetization” and is followed by number indicating a peak of alternating magnetic field in units of milli Tesla [mT]. Columns C, D, E: Marked as “x”, “y”, “z”. Detection of the three perpendicular magnetic components characterizing the magnetic strength of the sample. To get the proper units of this measurements, the number needs to be multiplied by power of 10 listed in column F. The magnitude of the magnetic vector is listed in column R (M [A/m]). Column S shows normalized magnetic vector amplitudes to its initial value listed before the onset of demagnetization. Column T shows a level of demagnetization by alternating magnetic field in [mT]. Column U shows magnetic efficiency obtained by ratio of non-saturated and saturated values demagnetized to specific level of alternating magnetic field, labelled as “REM”. Column V shows estimating paleofield assuming that the magnetic carrier in this sample is magnetite (used multiplication factor of 2800^1^).

Table S8: Magnetic data for sample 15B, when demagnetized by alternating magnetic field. Column A: Marked as “ID”. Identification name of the sample. When the name is followed by letters “PULS”, the sample’s magnetic state was reset be exposing to 1T pulse magnetic field and demagnetized in the same set of steps as prior to magnetic field exposure. Column B: Marked as: ”AF[mT]”. It has alphanumerical label, where AD stands for “alternating demagnetization” and is followed by number indicating a peak of alternating magnetic field in units of milli Tesla [mT]. Columns C, D, E: Marked as “x”, “y”, “z”. Detection of the three perpendicular magnetic components characterizing the magnetic strength of the sample. To get the proper units of this measurements, the number needs to be multiplied by power of 10 listed in column F. The magnitude of the magnetic vector is listed in column R (M [A/m]). Column S shows normalized magnetic vector amplitudes to its initial value listed before the onset of demagnetization. Column T shows a level of demagnetization by alternating magnetic field in [mT]. Column U shows magnetic efficiency obtained by ratio of non-saturated and saturated values demagnetized to specific level of alternating magnetic field, labelled as “REM”. Column V shows estimating paleofield assuming that the magnetic carrier in this sample is magnetite (used multiplication factor of 2800^1^).

Table S9: Magnetic data for sample 15C, when demagnetized by alternating magnetic field. Column A: Marked as “ID”. Identification name of the sample. When the name is followed by letters “PULS”, the sample’s magnetic state was reset be exposing to 1T pulse magnetic field and demagnetized in the same set of steps as prior to magnetic field exposure. Column B: Marked as: ”AF[mT]”. It has alphanumerical label, where AD stands for “alternating demagnetization” and is followed by number indicating a peak of alternating magnetic field in units of milli Tesla [mT]. Columns C, D, E: Marked as “x”, “y”, “z”. Detection of the three perpendicular magnetic components characterizing the magnetic strength of the sample. To get the proper units of this measurements, the number needs to be multiplied by power of 10 listed in column F. The magnitude of the magnetic vector is listed in column R (M [A/m]). Column S shows normalized magnetic vector amplitudes to its initial value listed before the onset of demagnetization. Column T shows a level of demagnetization by alternating magnetic field in [mT]. Column U shows magnetic efficiency obtained by ratio of non-saturated and saturated values demagnetized to specific level of alternating magnetic field, labelled as “REM”. Column V shows estimating paleofield assuming that the magnetic carrier in this sample is magnetite (used multiplication factor of 2800^1^).

Table S10: Magnetic data for sample 15D, when demagnetized by alternating magnetic field. Column A: Marked as “ID”. Identification name of the sample. When the name is followed by letters “PULS”, the sample’s magnetic state was reset be exposing to 1T pulse magnetic field and demagnetized in the same set of steps as prior to magnetic field exposure. Column B: Marked as: ”AF[mT]”. It has alphanumerical label, where AD stands for “alternating demagnetization” and is followed by number indicating a peak of alternating magnetic field in units of milli Tesla [mT]. Columns C, D, E: Marked as “x”, “y”, “z”. Detection of the three perpendicular magnetic components characterizing the magnetic strength of the sample. To get the proper units of this measurements, the number needs to be multiplied by power of 10 listed in column F. The magnitude of the magnetic vector is listed in column R (M [A/m]). Column S shows normalized magnetic vector amplitudes to its initial value listed before the onset of demagnetization. Column T shows a level of demagnetization by alternating magnetic field in [mT]. Column U shows magnetic efficiency obtained by ratio of non-saturated and saturated values demagnetized to specific level of alternating magnetic field, labelled as “REM”. Column V shows estimating paleofield assuming that the magnetic carrier in this sample is magnetite (used multiplication factor of 2800^1^).

Table S11: Magnetic data for sample 21D, when demagnetized by alternating magnetic field. Column A: Marked as “ID”. Identification name of the sample. When the name is followed by letters “PULS”, the sample’s magnetic state was reset be exposing to 1T pulse magnetic field and demagnetized in the same set of steps as prior to magnetic field exposure. Column B: Marked as: ”AF[mT]”. It has alphanumerical label, where AD stands for “alternating demagnetization” and is followed by number indicating a peak of alternating magnetic field in units of milli Tesla [mT]. Columns C, D, E: Marked as “x”, “y”, “z”. Detection of the three perpendicular magnetic components characterizing the magnetic strength of the sample. To get the proper units of this measurements, the number needs to be multiplied by power of 10 listed in column F. The magnitude of the magnetic vector is listed in column R (M [A/m]). Column S shows normalized magnetic vector amplitudes to its initial value listed before the onset of demagnetization. Column T shows a level of demagnetization by alternating magnetic field in [mT]. Column U shows magnetic efficiency obtained by ratio of non-saturated and saturated values demagnetized to specific level of alternating magnetic field, labelled as “REM”. Column V shows estimating paleofield assuming that the magnetic carrier in this sample is magnetite (used multiplication factor of 2800^1^).

Table S12: Magnetic data for sample 22D, when demagnetized by alternating magnetic field. Column A: Marked as “ID”. Identification name of the sample. When the name is followed by letters “PULS”, the sample’s magnetic state was reset be exposing to 1T pulse magnetic field and demagnetized in the same set of steps as prior to magnetic field exposure. Column B: Marked as: ”AF[mT]”. It has alphanumerical label, where AD stands for “alternating demagnetization” and is followed by number indicating a peak of alternating magnetic field in units of milli Tesla [mT]. Columns C, D, E: Marked as “x”, “y”, “z”. Detection of the three perpendicular magnetic components characterizing the magnetic strength of the sample. To get the proper units of this measurements, the number needs to be multiplied by power of 10 listed in column F. The magnitude of the magnetic vector is listed in column R (M [A/m]). Column S shows normalized magnetic vector amplitudes to its initial value listed before the onset of demagnetization. Column T shows a level of demagnetization by alternating magnetic field in [mT]. Column U shows magnetic efficiency obtained by ratio of non-saturated and saturated values demagnetized to specific level of alternating magnetic field, labelled as “REM”. Column V shows estimating paleofield assuming that the magnetic carrier in this sample is magnetite (used multiplication factor of 2800^1^).

Table S13: Magnetic data for sample 24C, when demagnetized by alternating magnetic field. Column A: Marked as “ID”. Identification name of the sample. When the name is followed by letters “PULS”, the sample’s magnetic state was reset be exposing to 1T pulse magnetic field and demagnetized in the same set of steps as prior to magnetic field exposure. Column B: Marked as: ”AF[mT]”. It has alphanumerical label, where AD stands for “alternating demagnetization” and is followed by number indicating a peak of alternating magnetic field in units of milli Tesla [mT]. Columns C, D, E: Marked as “x”, “y”, “z”. Detection of the three perpendicular magnetic components characterizing the magnetic strength of the sample. To get the proper units of this measurements, the number needs to be multiplied by power of 10 listed in column F. The magnitude of the magnetic vector is listed in column R (M [A/m]). Column S shows normalized magnetic vector amplitudes to its initial value listed before the onset of demagnetization. Column T shows a level of demagnetization by alternating magnetic field in [mT]. Column U shows magnetic efficiency obtained by ratio of non-saturated and saturated values demagnetized to specific level of alternating magnetic field, labelled as “REM”. Column V shows estimating paleofield assuming that the magnetic carrier in this sample is magnetite (used multiplication factor of 2800^1^).

Table S14: Magnetic data for sample 25A, when demagnetized by alternating magnetic field. Column A: Marked as “ID”. Identification name of the sample. When the name is followed by letters “PULS”, the sample’s magnetic state was reset be exposing to 1T pulse magnetic field and demagnetized in the same set of steps as prior to magnetic field exposure. Column B: Marked as: ”AF[mT]”. It has alphanumerical label, where AD stands for “alternating demagnetization” and is followed by number indicating a peak of alternating magnetic field in units of milli Tesla [mT]. Columns C, D, E: Marked as “x”, “y”, “z”. Detection of the three perpendicular magnetic components characterizing the magnetic strength of the sample. To get the proper units of this measurements, the number needs to be multiplied by power of 10 listed in column F. The magnitude of the magnetic vector is listed in column R (M [A/m]). Column S shows normalized magnetic vector amplitudes to its initial value listed before the onset of demagnetization. Column T shows a level of demagnetization by alternating magnetic field in [mT]. Column U shows magnetic efficiency obtained by ratio of non-saturated and saturated values demagnetized to specific level of alternating magnetic field, labelled as “REM”. Column V shows estimating paleofield assuming that the magnetic carrier in this sample is magnetite (used multiplication factor of 2800^1^).

Table S15: Magnetic data for sample 25B, when demagnetized by alternating magnetic field. Column A: Marked as “ID”. Identification name of the sample. When the name is followed by letters “PULS”, the sample’s magnetic state was reset be exposing to 1T pulse magnetic field and demagnetized in the same set of steps as prior to magnetic field exposure. Column B: Marked as: ”AF[mT]”. It has alphanumerical label, where AD stands for “alternating demagnetization” and is followed by number indicating a peak of alternating magnetic field in units of milli Tesla [mT]. Columns C, D, E: Marked as “x”, “y”, “z”. Detection of the three perpendicular magnetic components characterizing the magnetic strength of the sample. To get the proper units of this measurements, the number needs to be multiplied by power of 10 listed in column F. The magnitude of the magnetic vector is listed in column R (M [A/m]). Column S shows normalized magnetic vector amplitudes to its initial value listed before the onset of demagnetization. Column T shows a level of demagnetization by alternating magnetic field in [mT]. Column U shows magnetic efficiency obtained by ratio of non-saturated and saturated values demagnetized to specific level of alternating magnetic field, labelled as “REM”. Column V shows estimating paleofield assuming that the magnetic carrier in this sample is magnetite (used multiplication factor of 2800^1^).

Table S16: Magnetic data for sample 31D, when demagnetized by alternating magnetic field. Column A: Marked as “ID”. Identification name of the sample. When the name is followed by letters “PULS”, the sample’s magnetic state was reset be exposing to 1T pulse magnetic field and demagnetized in the same set of steps as prior to magnetic field exposure. Column B: Marked as: ”AF[mT]”. It has alphanumerical label, where AD stands for “alternating demagnetization” and is followed by number indicating a peak of alternating magnetic field in units of milli Tesla [mT]. Columns C, D, E: Marked as “x”, “y”, “z”. Detection of the three perpendicular magnetic components characterizing the magnetic strength of the sample. To get the proper units of this measurements, the number needs to be multiplied by power of 10 listed in column F. The magnitude of the magnetic vector is listed in column R (M [A/m]). Column S shows normalized magnetic vector amplitudes to its initial value listed before the onset of demagnetization. Column T shows a level of demagnetization by alternating magnetic field in [mT]. Column U shows magnetic efficiency obtained by ratio of non-saturated and saturated values demagnetized to specific level of alternating magnetic field, labelled as “REM”. Column V shows estimating paleofield assuming that the magnetic carrier in this sample is magnetite (used multiplication factor of 2800^1^).

Table S17: Magnetic data for sample 34A, when demagnetized by alternating magnetic field. Column A: Marked as “ID”. Identification name of the sample. When the name is followed by letters “PULS”, the sample’s magnetic state was reset be exposing to 1T pulse magnetic field and demagnetized in the same set of steps as prior to magnetic field exposure. Column B: Marked as: ”AF[mT]”. It has alphanumerical label, where AD stands for “alternating demagnetization” and is followed by number indicating a peak of alternating magnetic field in units of milli Tesla [mT]. Columns C, D, E: Marked as “x”, “y”, “z”. Detection of the three perpendicular magnetic components characterizing the magnetic strength of the sample. To get the proper units of this measurements, the number needs to be multiplied by power of 10 listed in column F. The magnitude of the magnetic vector is listed in column R (M [A/m]). Column S shows normalized magnetic vector amplitudes to its initial value listed before the onset of demagnetization. Column T shows a level of demagnetization by alternating magnetic field in [mT]. Column U shows magnetic efficiency obtained by ratio of non-saturated and saturated values demagnetized to specific level of alternating magnetic field, labelled as “REM”. Column V shows estimating paleofield assuming that the magnetic carrier in this sample is magnetite (used multiplication factor of 2800^1^).

Table S18: Magnetic data for sample 34B, when demagnetized by alternating magnetic field. Column A: Marked as “ID”. Identification name of the sample. When the name is followed by letters “PULS”, the sample’s magnetic state was reset be exposing to 1T pulse magnetic field and demagnetized in the same set of steps as prior to magnetic field exposure. Column B: Marked as: ”AF[mT]”. It has alphanumerical label, where AD stands for “alternating demagnetization” and is followed by number indicating a peak of alternating magnetic field in units of milli Tesla [mT]. Columns C, D, E: Marked as “x”, “y”, “z”. Detection of the three perpendicular magnetic components characterizing the magnetic strength of the sample. To get the proper units of this measurements, the number needs to be multiplied by power of 10 listed in column F. The magnitude of the magnetic vector is listed in column R (M [A/m]). Column S shows normalized magnetic vector amplitudes to its initial value listed before the onset of demagnetization. Column T shows a level of demagnetization by alternating magnetic field in [mT]. Column U shows magnetic efficiency obtained by ratio of non-saturated and saturated values demagnetized to specific level of alternating magnetic field, labelled as “REM”. Column V shows estimating paleofield assuming that the magnetic carrier in this sample is magnetite (used multiplication factor of 2800^1^).

Table S19: Magnetic data for sample 35A, when demagnetized by alternating magnetic field. Column A: Marked as “ID”. Identification name of the sample. When the name is followed by letters “PULS”, the sample’s magnetic state was reset be exposing to 1T pulse magnetic field and demagnetized in the same set of steps as prior to magnetic field exposure. Column B: Marked as: ”AF[mT]”. It has alphanumerical label, where AD stands for “alternating demagnetization” and is followed by number indicating a peak of alternating magnetic field in units of milli Tesla [mT]. Columns C, D, E: Marked as “x”, “y”, “z”. Detection of the three perpendicular magnetic components characterizing the magnetic strength of the sample. To get the proper units of this measurements, the number needs to be multiplied by power of 10 listed in column F. The magnitude of the magnetic vector is listed in column R (M [A/m]). Column S shows normalized magnetic vector amplitudes to its initial value listed before the onset of demagnetization. Column T shows a level of demagnetization by alternating magnetic field in [mT]. Column U shows magnetic efficiency obtained by ratio of non-saturated and saturated values demagnetized to specific level of alternating magnetic field, labelled as “REM”. Column V shows estimating paleofield assuming that the magnetic carrier in this sample is magnetite (used multiplication factor of 2800^1^).

Table S20: Magnetic data for sample 35B, when demagnetized by alternating magnetic field. Column A: Marked as “ID”. Identification name of the sample. When the name is followed by letters “PULS”, the sample’s magnetic state was reset be exposing to 1T pulse magnetic field and demagnetized in the same set of steps as prior to magnetic field exposure. Column B: Marked as: ”AF[mT]”. It has alphanumerical label, where AD stands for “alternating demagnetization” and is followed by number indicating a peak of alternating magnetic field in units of milli Tesla [mT]. Columns C, D, E: Marked as “x”, “y”, “z”. Detection of the three perpendicular magnetic components characterizing the magnetic strength of the sample. To get the proper units of this measurements, the number needs to be multiplied by power of 10 listed in column F. The magnitude of the magnetic vector is listed in column R (M [A/m]). Column S shows normalized magnetic vector amplitudes to its initial value listed before the onset of demagnetization. Column T shows a level of demagnetization by alternating magnetic field in [mT]. Column U shows magnetic efficiency obtained by ratio of non-saturated and saturated values demagnetized to specific level of alternating magnetic field, labelled as “REM”. Column V shows estimating paleofield assuming that the magnetic carrier in this sample is magnetite (used multiplication factor of 2800^1^).

Table S21: Magnetic data for sample 35C, when demagnetized by alternating magnetic field. Column A: Marked as “ID”. Identification name of the sample. When the name is followed by letters “PULS”, the sample’s magnetic state was reset be exposing to 1T pulse magnetic field and demagnetized in the same set of steps as prior to magnetic field exposure. Column B: Marked as: ”AF[mT]”. It has alphanumerical label, where AD stands for “alternating demagnetization” and is followed by number indicating a peak of alternating magnetic field in units of milli Tesla [mT]. Columns C, D, E: Marked as “x”, “y”, “z”. Detection of the three perpendicular magnetic components characterizing the magnetic strength of the sample. To get the proper units of this measurements, the number needs to be multiplied by power of 10 listed in column F. The magnitude of the magnetic vector is listed in column R (M [A/m]). Column S shows normalized magnetic vector amplitudes to its initial value listed before the onset of demagnetization. Column T shows a level of demagnetization by alternating magnetic field in [mT]. Column U shows magnetic efficiency obtained by ratio of non-saturated and saturated values demagnetized to specific level of alternating magnetic field, labelled as “REM”. Column V shows estimating paleofield assuming that the magnetic carrier in this sample is magnetite (used multiplication factor of 2800^1^).

Table S22: Magnetic data for sample 41B, when demagnetized by alternating magnetic field. Column A: Marked as “ID”. Identification name of the sample. When the name is followed by letters “PULS”, the sample’s magnetic state was reset be exposing to 1T pulse magnetic field and demagnetized in the same set of steps as prior to magnetic field exposure. Column B: Marked as: ”AF[mT]”. It has alphanumerical label, where AD stands for “alternating demagnetization” and is followed by number indicating a peak of alternating magnetic field in units of milli Tesla [mT]. Columns C, D, E: Marked as “x”, “y”, “z”. Detection of the three perpendicular magnetic components characterizing the magnetic strength of the sample. To get the proper units of this measurements, the number needs to be multiplied by power of 10 listed in column F. The magnitude of the magnetic vector is listed in column R (M [A/m]). Column S shows normalized magnetic vector amplitudes to its initial value listed before the onset of demagnetization. Column T shows a level of demagnetization by alternating magnetic field in [mT]. Column U shows magnetic efficiency obtained by ratio of non-saturated and saturated values demagnetized to specific level of alternating magnetic field, labelled as “REM”. Column V shows estimating paleofield assuming that the magnetic carrier in this sample is magnetite (used multiplication factor of 2800^1^).

Table S23: Magnetic data for sample 41C, when demagnetized by alternating magnetic field. Column A: Marked as “ID”. Identification name of the sample. When the name is followed by letters “PULS”, the sample’s magnetic state was reset be exposing to 1T pulse magnetic field and demagnetized in the same set of steps as prior to magnetic field exposure. Column B: Marked as: ”AF[mT]”. It has alphanumerical label, where AD stands for “alternating demagnetization” and is followed by number indicating a peak of alternating magnetic field in units of milli Tesla [mT]. Columns C, D, E: Marked as “x”, “y”, “z”. Detection of the three perpendicular magnetic components characterizing the magnetic strength of the sample. To get the proper units of this measurements, the number needs to be multiplied by power of 10 listed in column F. The magnitude of the magnetic vector is listed in column R (M [A/m]). Column S shows normalized magnetic vector amplitudes to its initial value listed before the onset of demagnetization. Column T shows a level of demagnetization by alternating magnetic field in [mT]. Column U shows magnetic efficiency obtained by ratio of non-saturated and saturated values demagnetized to specific level of alternating magnetic field, labelled as “REM”. Column V shows estimating paleofield assuming that the magnetic carrier in this sample is magnetite (used multiplication factor of 2800^1^).

Table S24: Magnetic data for sample 41D, when demagnetized by alternating magnetic field. Column A: Marked as “ID”. Identification name of the sample. When the name is followed by letters “PULS”, the sample’s magnetic state was reset be exposing to 1T pulse magnetic field and demagnetized in the same set of steps as prior to magnetic field exposure. Column B: Marked as: ”AF[mT]”. It has alphanumerical label, where AD stands for “alternating demagnetization” and is followed by number indicating a peak of alternating magnetic field in units of milli Tesla [mT]. Columns C, D, E: Marked as “x”, “y”, “z”. Detection of the three perpendicular magnetic components characterizing the magnetic strength of the sample. To get the proper units of this measurements, the number needs to be multiplied by power of 10 listed in column F. The magnitude of the magnetic vector is listed in column R (M [A/m]). Column S shows normalized magnetic vector amplitudes to its initial value listed before the onset of demagnetization. Column T shows a level of demagnetization by alternating magnetic field in [mT]. Column U shows magnetic efficiency obtained by ratio of non-saturated and saturated values demagnetized to specific level of alternating magnetic field, labelled as “REM”. Column V shows estimating paleofield assuming that the magnetic carrier in this sample is magnetite (used multiplication factor of 2800^1^).

Table S25: Magnetic data for sample 43D, when demagnetized by alternating magnetic field. Column A: Marked as “ID”. Identification name of the sample. When the name is followed by letters “PULS”, the sample’s magnetic state was reset be exposing to 1T pulse magnetic field and demagnetized in the same set of steps as prior to magnetic field exposure. Column B: Marked as: ”AF[mT]”. It has alphanumerical label, where AD stands for “alternating demagnetization” and is followed by number indicating a peak of alternating magnetic field in units of milli Tesla [mT]. Columns C, D, E: Marked as “x”, “y”, “z”. Detection of the three perpendicular magnetic components characterizing the magnetic strength of the sample. To get the proper units of this measurements, the number needs to be multiplied by power of 10 listed in column F. The magnitude of the magnetic vector is listed in column R (M [A/m]). Column S shows normalized magnetic vector amplitudes to its initial value listed before the onset of demagnetization. Column T shows a level of demagnetization by alternating magnetic field in [mT]. Column U shows magnetic efficiency obtained by ratio of non-saturated and saturated values demagnetized to specific level of alternating magnetic field, labelled as “REM”. Column V shows estimating paleofield assuming that the magnetic carrier in this sample is magnetite (used multiplication factor of 2800^1^).

Table S26: Magnetic data for sample 53D, when demagnetized by alternating magnetic field. Column A: Marked as “ID”. Identification name of the sample. When the name is followed by letters “PULS”, the sample’s magnetic state was reset be exposing to 1T pulse magnetic field and demagnetized in the same set of steps as prior to magnetic field exposure. Column B: Marked as: ”AF[mT]”. It has alphanumerical label, where AD stands for “alternating demagnetization” and is followed by number indicating a peak of alternating magnetic field in units of milli Tesla [mT]. Columns C, D, E: Marked as “x”, “y”, “z”. Detection of the three perpendicular magnetic components characterizing the magnetic strength of the sample. To get the proper units of this measurements, the number needs to be multiplied by power of 10 listed in column F. The magnitude of the magnetic vector is listed in column R (M [A/m]). Column S shows normalized magnetic vector amplitudes to its initial value listed before the onset of demagnetization. Column T shows a level of demagnetization by alternating magnetic field in [mT]. Column U shows magnetic efficiency obtained by ratio of non-saturated and saturated values demagnetized to specific level of alternating magnetic field, labelled as “REM”. Column V shows estimating paleofield assuming that the magnetic carrier in this sample is magnetite (used multiplication factor of 2800^1^).

Table S27: Magnetic data for sample 51C, when demagnetized by alternating magnetic field. Column A: Marked as “ID”. Identification name of the sample. When the name is followed by letters “PULS”, the sample’s magnetic state was reset be exposing to 1T pulse magnetic field and demagnetized in the same set of steps as prior to magnetic field exposure. Column B: Marked as: ”AF[mT]”. It has alphanumerical label, where AD stands for “alternating demagnetization” and is followed by number indicating a peak of alternating magnetic field in units of milli Tesla [mT]. Columns C, D, E: Marked as “x”, “y”, “z”. Detection of the three perpendicular magnetic components characterizing the magnetic strength of the sample. To get the proper units of this measurements, the number needs to be multiplied by power of 10 listed in column F. The magnitude of the magnetic vector is listed in column R (M [A/m]). Column S shows normalized magnetic vector amplitudes to its initial value listed before the onset of demagnetization. Column T shows a level of demagnetization by alternating magnetic field in [mT]. Column U shows magnetic efficiency obtained by ratio of non-saturated and saturated values demagnetized to specific level of alternating magnetic field, labelled as “REM”. Column V shows estimating paleofield assuming that the magnetic carrier in this sample is magnetite (used multiplication factor of 2800^1^).

Table S28: Magnetic data for sample 29A, when demagnetized by alternating magnetic field. Column A: Marked as “ID”. Identification name of the sample. When the name is followed by letters “PULSE”, the sample’s magnetic state was reset be exposing to 1T pulse magnetic field and demagnetized in the same set of steps as prior to magnetic field exposure. Column B: Marked as: ”AF[mT]”. It has alphanumerical label, where AD stands for “alternating demagnetization” and is followed by number indicating a peak of alternating magnetic field in units of milli Tesla [mT]. Columns C, D, E: Marked as “x”, “y”, “z”. Detection of the three perpendicular magnetic components characterizing the magnetic strength of the sample. To get the proper units of this measurements, the number needs to be multiplied by power of 10 listed in column F. The magnitude of the magnetic vector is listed in column R (M [A/m]). Column S shows normalized magnetic vector amplitudes to its initial value listed before the onset of demagnetization. Column T shows a level of demagnetization by alternating magnetic field in [mT]. Column U shows magnetic efficiency obtained by ratio of non-saturated and saturated values demagnetized to specific level of alternating magnetic field, labelled as “REM”. Column V shows estimating paleofield assuming that the magnetic carrier in this sample is magnetite (used multiplication factor of 2800^1^).

Table S29: Magnetic data for sample 29B, when demagnetized by alternating magnetic field. Column A: Marked as “ID”. Identification name of the sample. When the name is followed by letters “PULSE”, the sample’s magnetic state was reset be exposing to 1T pulse magnetic field and demagnetized in the same set of steps as prior to magnetic field exposure. Column B: Marked as: ”AF[mT]”. It has alphanumerical label, where AD stands for “alternating demagnetization” and is followed by number indicating a peak of alternating magnetic field in units of milli Tesla [mT]. Columns C, D, E: Marked as “x”, “y”, “z”. Detection of the three perpendicular magnetic components characterizing the magnetic strength of the sample. To get the proper units of this measurements, the number needs to be multiplied by power of 10 listed in column F. The magnitude of the magnetic vector is listed in column R (M [A/m]). Column S shows normalized magnetic vector amplitudes to its initial value listed before the onset of demagnetization. Column T shows a level of demagnetization by alternating magnetic field in [mT]. Column U shows magnetic efficiency obtained by ratio of non-saturated and saturated values demagnetized to specific level of alternating magnetic field, labelled as “REM”. Column V shows estimating paleofield assuming that the magnetic carrier in this sample is magnetite (used multiplication factor of 2800^1^).

Table S30: Magnetic data for sample 31A, when demagnetized by alternating magnetic field. Column A: Marked as “ID”. Identification name of the sample. When the name is followed by letters “PULSE”, the sample’s magnetic state was reset be exposing to 1T pulse magnetic field and demagnetized in the same set of steps as prior to magnetic field exposure. Column B: Marked as: ”AF[mT]”. It has alphanumerical label, where AD stands for “alternating demagnetization” and is followed by number indicating a peak of alternating magnetic field in units of milli Tesla [mT]. Columns C, D, E: Marked as “x”, “y”, “z”. Detection of the three perpendicular magnetic components characterizing the magnetic strength of the sample. To get the proper units of this measurements, the number needs to be multiplied by power of 10 listed in column F. The magnitude of the magnetic vector is listed in column R (M [A/m]). Column S shows normalized magnetic vector amplitudes to its initial value listed before the onset of demagnetization. Column T shows a level of demagnetization by alternating magnetic field in [mT]. Column U shows magnetic efficiency obtained by ratio of non-saturated and saturated values demagnetized to specific level of alternating magnetic field, labelled as “REM”. Column V shows estimating paleofield assuming that the magnetic carrier in this sample is magnetite (used multiplication factor of 2800^1^).

Table S31: Magnetic data for sample 31B, when demagnetized by alternating magnetic field. Column A: Marked as “ID”. Identification name of the sample. When the name is followed by letters “PULSE”, the sample’s magnetic state was reset be exposing to 1T pulse magnetic field and demagnetized in the same set of steps as prior to magnetic field exposure. Column B: Marked as: ”AF[mT]”. It has alphanumerical label, where AD stands for “alternating demagnetization” and is followed by number indicating a peak of alternating magnetic field in units of milli Tesla [mT]. Columns C, D, E: Marked as “x”, “y”, “z”. Detection of the three perpendicular magnetic components characterizing the magnetic strength of the sample. To get the proper units of this measurements, the number needs to be multiplied by power of 10 listed in column F. The magnitude of the magnetic vector is listed in column R (M [A/m]). Column S shows normalized magnetic vector amplitudes to its initial value listed before the onset of demagnetization. Column T shows a level of demagnetization by alternating magnetic field in [mT]. Column U shows magnetic efficiency obtained by ratio of non-saturated and saturated values demagnetized to specific level of alternating magnetic field, labelled as “REM”. Column V shows estimating paleofield assuming that the magnetic carrier in this sample is magnetite (used multiplication factor of 2800^1^).

References:

1 Kletetschka, G. & Wieczorek, M. A. Fundamental Relations of Mineral Specific Magnetic Carriers for Paleointensity Determination. *Physics of the Earth and Planetary Interiors* **272**, 44-49, doi:<https://doi.org/10.1016/j.pepi.2017.09.008> (2017).
